# Studying Acetylation of Aconitase Isozymes by Genetic Code Expansion

**DOI:** 10.3389/fchem.2022.862483

**Published:** 2022-03-24

**Authors:** Jessica Araujo, Sara Ottinger, Sumana Venkat, Qinglei Gan, Chenguang Fan

**Affiliations:** ^1^ Cell and Molecular Biology Program, University of Arkansas, Fayetteville, AR, United States; ^2^ Department of Chemistry and Biochemistry, University of Arkansas, Fayetteville, AR, United States; ^3^ Children’s Research Institute, The University of Texas Southwestern Medical Center, Dallas, TX, United States

**Keywords:** lysine acetylation, aconitase, TCA cycle, genetic code expansion, deacetylase

## Abstract

Aconitase catalyzes the second reaction of the tricarboxylic acid cycle, the reversible conversion of citrate and isocitrate. *Escherichia coli* has two isoforms of aconitase, AcnA and AcnB. Acetylomic studies have identified acetylation at multiple lysine sites of both *E. coli* aconitase isozymes, but the impacts of acetylation on aconitases are unknown. In this study, we applied the genetic code expansion approach to produce 14 site-specifically acetylated aconitase variants. Enzyme assays and kinetic analyses showed that acetylation of AcnA K684 decreased the enzyme activity, while acetylation of AcnB K567 increased the enzyme activity. Further *in vitro* acetylation and deacetylation assays were performed, which indicated that both aconitase isozymes could be acetylated by acetyl-phosphate chemically, and be deacetylated by the CobB deacetylase at most lysine sites. Through this study, we have demonstrated practical applications of genetic code expansion in acetylation studies.

## Introduction

**SCHEME 1:**
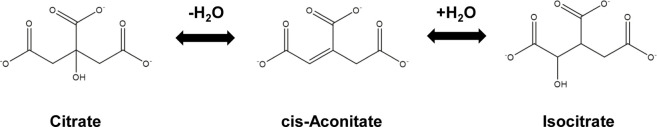
The reaction catalyzed by aconitase.

Aconitase catalyzes the reversible conversion of citrate and isocitrate in the tricarboxylic acid (**TCA**) and glyoxylate cycles (Scheme 1). It is an iron-sulfur (**Fe-S**) enzyme (Beinert et al., 1996). Depending on the state of the Fe-S cluster, aconitase has three forms: the active (4Fe-4S) form, the inactive (3Fe-4S) form, and the apo-enzyme form. The (4Fe-4S) cluster is sensitive to reactive oxygen species (**ROS**) and iron depletion which impair aconitase activities, so aconitases are widely used as biomarkers for oxidative stress and intracellular sensors of iron and redox states (Gardner, 2002; Castro et al., 2019). In *E. coli* cells, there are two aconitase isozymes, AcnA and AcnB (Gruer and Guest, 1994). Enzymological and regulatory analyses indicated that AcnB is the major TCA enzyme expressed during the exponential phase while AcnA is synthesized during the stationary phase or under stress conditions (Cunningham et al., 1997). Inactivated by ROS or iron depletion, both AcnA and AcnB apo-enzymes can bind to 3′-untranslated region of *acnA* and *acnB* mRNAs to stabilize them and increase their own expression, mediating a post-transcriptional positive autoregulation (Tang and Guest, 1999). Furthermore, aconitases are also regulated by post-translational modifications (**PTM**s), mostly oxidation, nitrosylation, and thiolation of cysteine residues around the Fe-S cluster (Lushchak et al., 2014). Recently, a number of acetylated lysine residues have been identified in aconitase isozymes of both mammals and bacteria (Xu et al., 2017). Two studies on mammalian mitochondrial aconitase (**mAcn**) indicated that acetylation can increase its enzyme activity. By acetylating mitochondria *in vitro* with acetic anhydride or acetyl-CoA and increasing the acetylation level in mice with high-fat diet, it was found that mAcn with higher acetylation levels had increased activities. Sequence alignment and structural modeling suggested the acetylation of the conserved K144 residue could induce such enhancement (Fernandes et al., 2015). In another study, K258 of mAcn was identified to be acetylated in human prostate cancer cells by mass spectrometry analysis. While replacing K258 with arginine, such acetylation-deficient variant had decrease enzyme activities. The deacetylase SIRT3 deacetylates mAcn and decreases its enzyme activity (Sawant Dessai et al., 2021). However, both *E. coli* AcnA and AcnB have low sequence identities with mammalian mAcn, so the impacts of acetylation on aconitase isozymes in *E. coli* remain unknown.

The classic approach to study lysine acetylation is to use glutamine (KQ mutation) as a mimic of acetyllysine. However, this method undermines the structural difference between glutamine and acetyllysine. The side chain of glutamine residue is ∼4 Å shorter than acetyllysine, so it may not reflect the real impacts of lysine acetylation. Indeed, our previous study on lysine acetylation of isocitrate dehydrogenase compared its activity with KQ mutations and real acetylated lysine residues, showing that at some acetylation sites the KQ mutation method derived different or even opposite conclusions (Venkat et al., 2018). To overcome this problem, the genetic code expansion technique has been used to generate site-specifically acetylated enzyme variants. This technique introduces an aminoacyl-tRNA synthetase which has been engineered to recognize acetyllysine and a tRNA which can decode a stop codon (UAG) as acetyllysine to produce site-specifically and purely acetylated proteins (Neumann et al., 2009). In this work, we used this approach to study lysine acetylation of aconitase isozymes, demonstrating a practical application of genetic code expansion in protein PTM studies.

## Materials and Methods

### General Molecular Biology and Protein Analyses

Chemicals were purchased from VWR International (Radnor, PA, United States) or Chem-Impex International (Wood Dale, IL, United States). Plasmid were constructed by the NEBuilder HiFi DNA Assembly Kit (New England Biolabs, Ipswich, MA, United States). Point mutations were generated by the Q5 Site-Directed Mutagenesis Kit (New England Biolabs). For western blotting, purified aconitase isozymes and their variants were separated on SDS PAGE gels and transferred to the PVDF membranes. The horseradish peroxidase (HRP)-conjugated acetyllysine antibody (Cell Signaling Technology, Danvers, MA, United States) was used as the primary antibody, and chemiluminescence for detection was generated by Pierce ECL Western Blotting substrates (Thermo Scientific, Waltham, MA, United States).

### Expression and Purification of Aconitases and Acetylated Variants

The gene of *acnA* or *acnB* or their mutants was cloned into the *pCDF-1b* plasmid (EMD Millipore, Burlington, MA, United States) with a C-terminal His_6_-tag, individually. Then it was transformed into BL21 (DE3) cells together with the acetyllysine incorporation system routinely used in our group (Venkat et al., 2017a). Cells was grown in 400 ml of LB medium with 100 μg/ml streptomycin, 50 μg/ml chloramphenicol, 10 mM acetyllysine, and 20 mM nicotinamine (NAM, the deacetylase inhibitor) at 37°C to OD 600 nm of 0.6–0.8, then 0.1 mM Isopropyl β-D-1-thiogalactopyranoside (IPTG) was added to induce protein expression. Cells were then incubated at 16°C for an additional 12 h and harvested by centrifugation at 4,000 × g for 20 min at 4°C. Cell pellets were suspended in 12 ml of 50 mM Tris (pH 7.8), 300 mM NaCl, 20 mM imidazole, 20 mM NAM, and 5 mM β-mercaptoethanol with cocktail protease inhibitors (Roche, Basel, Switzerland), and then broken by sonication. The crude extract was centrifuged at 20,000 × g for 30 min at 4°C. The soluble fraction was filtered through a 0.45-µm membrane and loaded onto a column containing 2 ml of Ni-NTA resin (Qiagen, Hilden, Germany). The column was then washed with 25 ml of 50 mM Tris (pH 7.8), 300 mM NaCl, 1 mM DTT, and 50 mM imidazole, and eluted with 2 ml of 50 mM Tris (pH 7.8), 300 mM NaCl, 1 mM DTT, and 200 mM imidazole. SDS-PAGE electrophoresis was performed to check the purity of aconitases and their variants. Western blotting and mass spectrometry were performed to confirm the incorporation of acetyllysine at correct sites.

### The Aconitase Activity Assay and Kinetic Analyses

Before enzyme activity assays, purified aconitases and their acetylated variants were reactivated by incubating with 1 mM (NH_4_)_2_Fe(SO_4_)_2_ and 5 mM DTT in 50 mM Tris (pH 8) for 30 min following previous protocols (Bradbury et al., 1996). Enzyme assays were performed with the commercial aconitase assay kit from BioAssay System (Hayward, CA). Briefly, it measures the isocitrate generated as a product of the aconitase reaction. The isocitrate is then oxidized producing NADPH and the oxidation product. The NADPH converts the dye to an intense violet color with an absorption maximum at 565 nm. The increase in absorbance at 565 nm is directly proportional to aconitase activity. To determine steady-state kinetic parameters, the concentration of the substrate citrate was varied from 0.1 to 50 mM. Kinetic parameters were calculated by non-liner regression with software Grafit (Erithacus Software).

### Mass Spectrometry Analyses

The LC-MS/MS analyses were performed by the Yale Keck Proteomics facility and followed the previous protocol (Venkat et al., 2018). Briefly, aconitases and their variants were separated by SDS-PAGE electrophoresis. Protein bands were cut and digested in gel by trypsin, and analyzed by LC-MS/MS on an LTQ Orbitrap XL equipped with a nanoACQUITY UPLC system. The Mascot search algorithm was used to search for the substitution of the lysine residue with acetyllysine. All the MS data have been deposited as ProteomeXchange dataset PXD031431.

### The *In Vitro* Acetylation Assay

The acetylation reaction was performed in the buffer of 50 mM Tris (pH 8.0), 0.1 mM EDTA, 1 mM DTT and 10 mM sodium butyrate, initiated by mixing 10 μg enzyme and 3 mM AcP in a total volume of 100 μL, and then incubated at 37°C for 1 h.

### The *In Vitro* Deacetylation Assay

The deacetylation reaction was performed in the buffer of 50 mM HEPES (pH 7.0), 5 mM MgCl_2_, 1.0 mM NAD^+^, and 1 mM DTT, initiated by mixing 10 μg enzyme and 10 μg purified CobB in a total volume of 100 μL, and then incubated at 37°C for 1 h.

## Results

### Generation of Site-Specifically Acetylated Aconitase Variants

Several quantitative acetylomic studies of *E. coli* cells have demonstrated that both aconitase isoenzymes have higher acetylation stoichiometry than many other *E. coli* proteins (Baeza et al., 2014; Meyer et al., 2016; Weinert et al., 2017). Thus, we aimed to identify the effects of acetylation on aconitase isoenzymes site-specifically. Although a series of acetylomic studies have been performed for *E. coli* cells, the sets of acetylation sites identified in aconitase isoenzymes do not overlap well because of differences in strains, growth media, and MS detection and resolutions (Yu et al., 2008; Zhang et al., 2009; Colak et al., 2013; Weinert et al., 2013; Zhang et al., 2013; Castano-Cerezo et al., 2014; Kuhn et al., 2014; AbouElfetouh et al., 2015; Schilling et al., 2015). To be feasible and avoid biased selection, we chose all the lysine residues identified to be acetylated by more than three independent acetylomic studies, which were K164, K342, K482, K684 of AcnA and K77, K373, K396, K407, K539, K559, K567, K728, K759, K835 of AcnB.

In this study, we utilized our optimized acetyllysine incorporation system to produce site-specifically acetylated aconitase variants at selected sites listed above individually (Venkat et al., 2017a). To minimize the non-specific acetylation of other lysine residues in aconitases, we used the BL21 (DE3) strain as the host cell line, which has a low level of acetylation globally (Weinert et al., 2013). Our previous studies on acetylation of malate dehydrogenase, isocitrate dehydrogenase, and citrate synthase have shown that those wild-type enzymes purified from BL21 (DE3) cells have low levels of non-specific acetylation (Venkat et al., 2017b; Venkat et al., 2018; Venkat et al., 2019). As expected, wild-type AcnA and AcnB overexpressed in BL21 (DE3) cells had no or very weak level of acetylation (Supplementary Figure S1). We fused the His_6_-tag to the C-terminus of aconitase variants for easy purification and to remove truncated proteins terminated at inserted UAG codons. All the purified acetylated aconitase variants had clear single bands in SDS-PAGE gels and were detected by the acetyllysine antibody in western blots (Supplementary Figure S1). The positions of acetyllysine incorporation were confirmed by LC-MS/MS analyses (Supplementary Figures S2–S15).

### The Site-Specific Effects of Lysine Acetylation on Aconitase Activities

**FIGURE 1:**
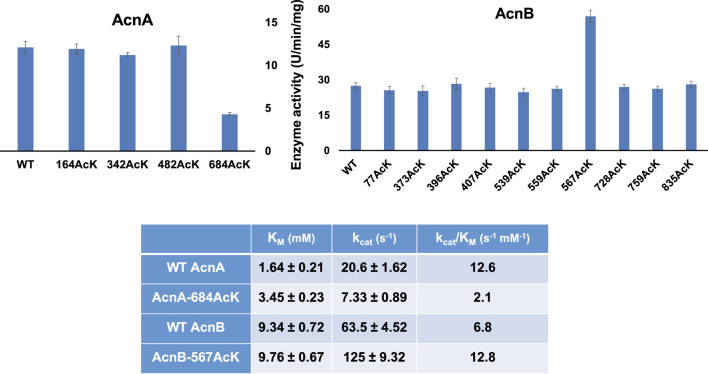
The enzyme activities and kinetic analyses of AcnA, AcnB and their acetylated variants. The **(A)** is enzyme activities measured by the commercial kit with 50 mM citrate as the substrate concentration. The **(B)** is the steady-state kinetic parameters. Kinetic parameters were calculated by non-liner regression with software Grafit. Mean values and standard deviations were calculated based on three replicates. The Student’s t test was used to analyze the significance of differences between activities of each acetylated variant and the corresponding WT enzyme.

First, we measured the enzyme activities of purified AcnA and AcnB as well as their site-specifically acetylated variants with the commercial kit individually (Figure 1). Acetylation of most lysine sites had no significant effects on aconitase activities (*p* = 0.726, 0.110, and 0.804 for AcnA variants at K164, K342 and K482, individually; *p* = 0.186, 0.198, 0.649, 0.580, 0.094, 0.257, 0.625, 0.257, 0.674 for AcnB variants at K77, K373, K396, K407, K539, K559, K728, K759, and K835, individually). There were only two variants which significantly affect aconitase activities (*p* < 0.001 for both AcnA K684 and AcnB K567). Interestingly, the impacts of acetylation on enzyme activities were different in aconitase isozymes. Acetylation of AcnA K684 decreased the activity by ∼3-fold while acetylation of AcnB K567 increased the activity by ∼2-fold.

To obtain insights into the impacts of acetylation on aconitase activities, we performed steady-state kinetic analyses of AcnA and AcnB as well as those two variants (AcnA-684AcK and AcnB-567AcK) which significantly affected activities (Figure 1). Acetylation of AcnA K684 impairs both the substrate binding and the turnover number while acetylation of AcnB K567 only enhances the turnover number.

### The Acetyl-Phosphate-Dependent Acetylation of Aconitase Isozymes

**FIGURE 2:**
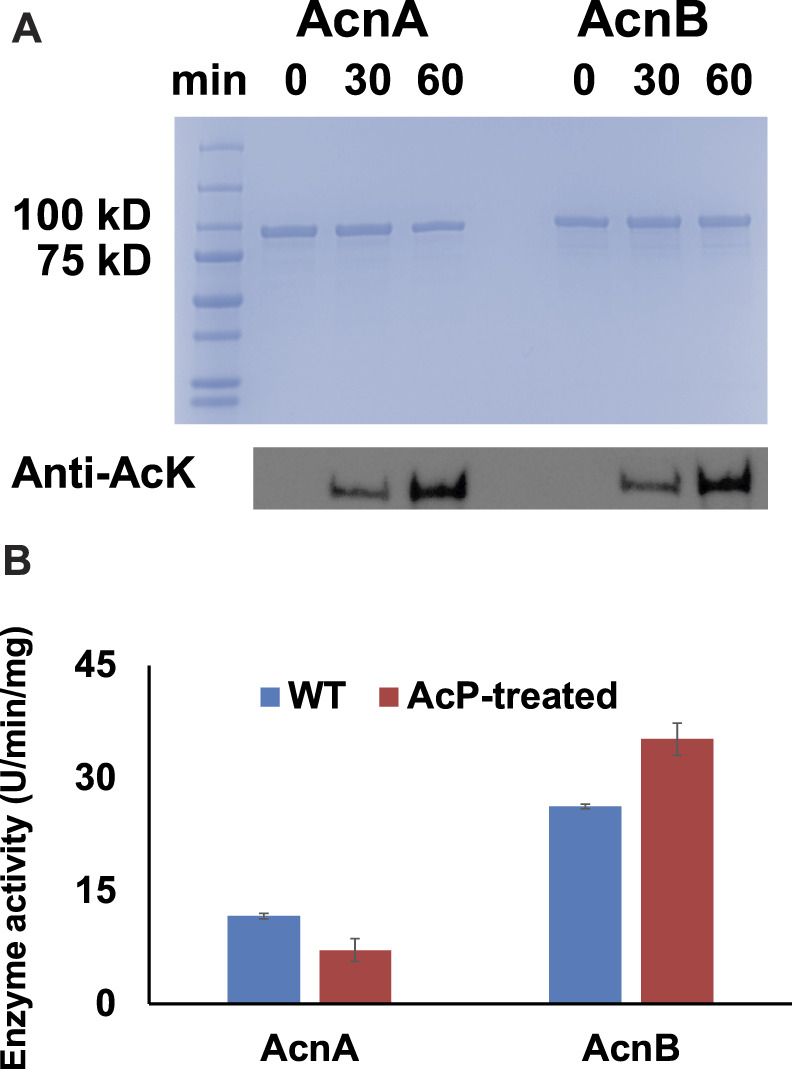
AcP-dependent acetylation of AcnA and AcnB. **(A)** SDS-PAGE and wester blots of purified WT AcnA and WT AcnB treated with AcP *in vitro*. 2 µg of proteins were loaded for each lane. The full image of western blots is in Supplementary Figure S16. **(B)** Enzyme activities of purified WT AcnA and WT AcnB before and after AcP-treatment. Mean values and standard deviations were calculated based on three replicates. The Student’s t test was used to analyze the significance of differences between activities of enzymes before and after AcP-treatment.

It is known that lysine acetylation in *E. coli* is mostly generated non-enzymatically with acetyl-phosphate (**AcP**) as the acetyl-donor while acetyl-CoA-dependent enzymatic acetylation only applies for a small portion of proteins (Weinert et al., 2013; Hentchel and Escalante-Semerena, 2015). Our previous studies also showed that AcP itself can acetylate several TCA cycle enzymes chemically (Venkat et al., 2017b; Venkat et al., 2018; Venkat et al., 2019). In this study, WT AcnA and AcnB expressed in BL21 (DE3) cells were purified and treated with 3 mM AcP *in vitro* which is the physiological concentration of AcP inside *E. coli* cells (Klein et al., 2007). Western blots showed that AcP acetylated both AcnA and AcnB in a time-dependent manner (Figure 2A). The activities of AcnA and AcnB before and after AcP-treatment were measured. Consistent with site-specific results above, acetylation of AcnA impaired its activity while acetylation of AcnB enhanced its activity (*p* < 0.001 for AcnA and *p* = 0.004 for AcnB) (Figure 2B). The impacts of acetylation by AcP-treatment were not as significant as the site-specific acetylation above, probably because AcP cannot acetylate lysine residues completely while purely acetylated variants were tested in above site-specific experiments.

Then we performed LC-MS/MS analyses to identify acetylation sites in AcnA and AcnB by AcP-treatment. Those 14 sites selected for site-specific tests above were all acetylated by *in vitro* AcP-treatment. Besides them, we also identified 12 additional acetylation sites in AcnA (K10, K16, K116, K257, K283, K391, K406, K453, K578, K758, K770, and, K823) and 11 additional acetylation sites in AcnB (K20, K64, K73, K135, K137, K144, K356, K387, K571, K613, and K722). Among them, K116, K257, K406, K453, K578, K823 of AcnA and K64, K137 of AcnB have not been identified to be acetylated in *E. coli* cells ever before, probably because acetylation of these sites has low stoichiometry and can be deacetylated easily in living cells. On the other hand, K18, K30, K71, K161, K460, K585, K832 of AcnA and K85, K110, K117, K221, K229, K267, K537, K588 of AcnB listed in the *E. coli* acetylation database (Xu et al., 2017) were not identified in our *in vitro* AcP-acetylation tests, implying that specific acetyltransferases or cofactors could be necessary for acetylation of these lysine sites in cells.

### The CobB-Dependent Deacetylation of Aconitase Isozymes

**FIGURE 3:**
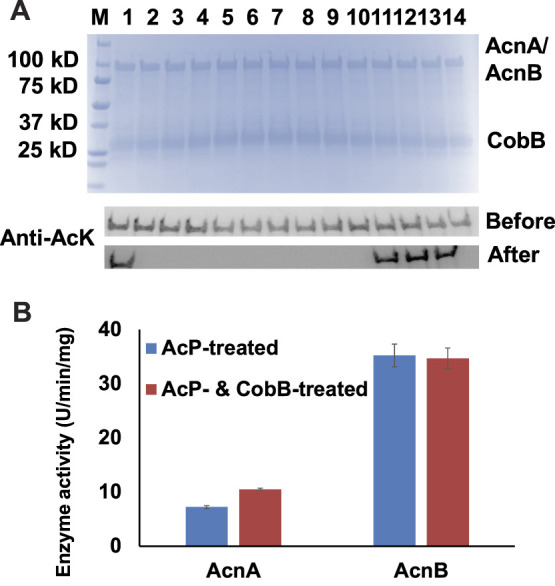
CobB-catalyzed deacetylation of AcnA and AcnB. **(A)** SDS-PAGE and wester blots of site-specifically acetylated AcnA and AcnB variants incubated with CobB *in vitro*. 2 µg of AcnA/AcnB and CobB were loaded for each lane. The full image of western blots is in Supplementary Figure S17. Samples from lane 1 to 4 are AcnA-164AcK, -342AcK, -482AcK, and -684AcK. Samples from lane 5 to 14 are AcnB-77AcK, 373-AcK, -396AcK, -407AcK, -539AcK, -559AcK, -567AcK, -728AcK, -759AcK, and -835AcK. **(B)** Enzyme activities of AcP-treated AcnA and AcnB before and after CobB-incubation. Mean values and standard deviations were calculated based on three replicates. The Student’s t test was used to analyze the significance of differences between activities of enzymes before and after CobB-treatment.

Acetylation of lysine residues is reversible, and the deacetylation of acetylated lysine residues is catalyzed by protein lysine deacetylases (**KDAC**). To date, CobB is still the only well-known KDAC in *E. coli* (Starai et al., 2002). Our previous studies showed that CobB can deacetylate acetylated lysine residues in several TCA cycle enzymes, but not for all the acetylation sites (Venkat et al., 2017b; Venkat et al., 2018; Venkat et al., 2019). In this study, we incubated those 14 site-specifically acetylated AcnA and AcnB variants with CobB, and used western blotting to determine the site specificity of CobB for AcnA and AcnB (Figure 3A). Most of acetylation sites were sensitive to CobB, while K164 of AcnA and K567, K728, K759 of AcnB were resistant to CobB.

Then we incubated AcP-treated AcnA and AcnB with CobB *in vitro*. After that, we measured the enzyme activities (Figure 3B). CobB-dependent acetylation restored AcnA activity (*p* < 0.001), but did not affect AcnB activity significantly (*p* = 0.775). Acetylation of K684 in AcnA decreases AcnA activity and K684 is sensitive to CobB, so deacetylation of K684 could restore its enzyme activity. On the other hand, acetylation of K567 in AcnB increases AcnB activity but K567 is resistant to CobB, so CobB-dependent deacetylation could not restore AcnB activity. These results also indicated that K684 of AcnA and K567 of AcnB are the two lysine residues whose acetylation affects their enzyme activities the most, which is consistent to our site-specific results above.

## Discussion

### The Effects of Acetylation on Aconitase Activities

**FIGURE 4:**
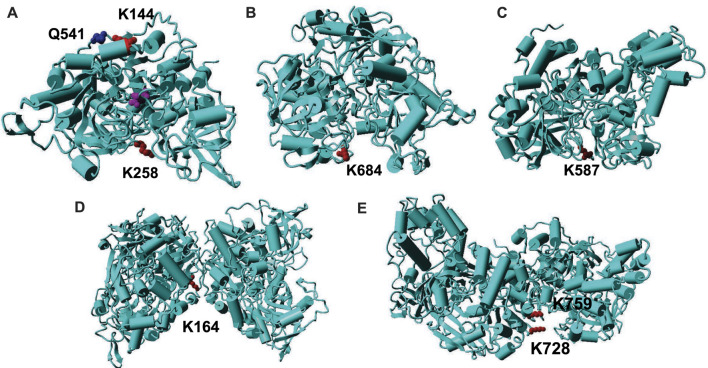
Mapping of acetylated lysine residues on the structures of AcnA and AcnB. **(A)** The crystal structure of human cAcn (PDB ID: 2B3Y). **(B)** The homology model of AcnA structure based on human cAcn. **(C)** The crystal structure of AcnB (PDB ID: 1L5J). **(D)** The homology model of AcnA dimer based on human cAcn. **(E)** The structure of AcnB dimer. The backbone of the protein is in cyan; lysine residues are in red; other key residues are in blue; the substrate is in purple.

Before our work, only two papers have reported acetylation of aconitase isoenzymes, which indicated that acetylation of K144 and K258 in mammalian mAcn enhances enzyme activities (Fernandes et al., 2015; Sawant Dessai et al., 2021). To find the structure-function relationships of aconitase acetylation, we mapped those two lysine residues onto the crystal structures of mAcn. Because there is no reported human mAcn structures, we used bovine mAcn as the model which has 96% sequence identify with human mAcn (Figure 4A). The active site is at the center of the enzyme (the purple molecule is the intermediate analog methyl-isocitrate). K144 is at the back of the active site. The previous study proposed that acetylation of K144 strengthens its interaction with the nearby Q541, hence inducing conformational changes to facilitate substrate binding (Fernandes et al., 2015). K258 is at the entrance of the active site, so its acetylation could also cause conformational changes to enhance enzyme activities.

The structure of human cytosolic Acn (**cAcn**) which has a 53% sequence identify with *E. coli* AcnA has been solved (Dupuy et al., 2006). The structure of *E. coli* AcnB has also been solved (Williams et al., 2002). Although both *E. coli* AcnA and AcnB have low sequence identifies with human mAcn (28 and 24%, respectively), the overall structures of different aconitases are very similar (Williams et al., 2002). Hence, we mapped K684 of AcnA and K567 of AcnB onto their structures. Both K684 of AcnA and K567 of AcnB are located at the entrance of the active sites of aconitase isozymes, but the orientations of two lysine residues are different (Figures 4B,C). K684 of AcnA faces outside the active site, and the addition of an acetyl group could limit the accessibility of the active sites, thus decreasing substrate binding and catalytic efficiency. On the other hand, K567 of AcnB faces inside the active site, and its acetylation may not affect the substrate binding. The additional acetyl group could fill the space of the active site to provide extra interactions to stabilize the intermediate, hence increasing the turnover of the reaction. Further structural studies such as crystalizing those site-specifically acetylated variants are necessary to identify the mechanisms of impacts from acetylation.

### The Sensitivities of Acetylated Lysine Residues in Aconitases Towards CobB Deacetylase

Our deacetylation tests showed that CobB can remove most of acetylated lysine resides in both AcnA and AcnB (Figure 3). Then we mapped those residues which are sensitive to CobB (Supplementary Figure S18). CobB-sensitive sites are all located at protein surface for easy CobB access, consistent with our previous studies on other TCA cycle enzymes (Venkat et al., 2017b; Venkat et al., 2018; Venkat et al., 2019). On the other hand, we also mapped those residues which are resistant to CobB. Both AcnA and AcnB form dimers in solutions (Jordan et al., 1999). We used human cAcn as the template to model the AcnA structure. K164 of AcnA is located at the interface of two subunits (Figure 4D). K728 and K759 of AcnB are also at the subunit interface (Figure 4E). Such steric hindrance limits the access of CobB for deacetylation. Although K567 is located at the entrance of the active site, the primary amine group points to interior of the active site (Figure 4C), and this orientation also limits the access of CobB for deacetylation. However, acetylation of K567 increases AcnB activity, so the acetylation of K567 should be regulated. Because we used purified CobB in *in vitro* experiments, some cofactors that are necessary for deacetylation of K567 may be missed in *in vitro* experiments. Furthermore, there could be other deacetylases in *E. coli* which can deacetylate those CobB-resistant positions in Acn. Indeed, besides CobB which belongs to class III lysine deacetylases, putative members of class I, II and IV lysine deacetylases have also been identified in bacteria (Hildmann et al., 2007).

In summary, we applied the genetic code expansion approach to generate site-specifically acetylated aconitase variants. With these purely acetylated variants on hand, we were able to identify key lysine residues, whose acetylation has significant impacts on enzyme functions. We could also test acetylation and deacetylation specificities by this approach. Thus, we have demonstrated practical applications of genetic code expansion in protein acetylation studies. Further studies will be implemented to investigate aconitase acetylation regulations in living cells. Together with genetic studies such as knocking out proteins involved in acetylation and deacetylation processes, the results from this work will provide solid evidence.

## Data Availability

The datasets presented in this study can be found in online repositories. The names of the repository/repositories and accession number(s) can be found below: MassIVE (Accession: MSV000088779), ProteomeXchange (Accession: PXD031431).
