# Supplementary material for: Studying Acetylation of Aconitase Isozymes by Genetic Code Expansion
Source: Front Chem. 2022 Mar 24;10:862483. doi: 10.3389/fchem.2022.862483 (PMC8987015; doi:10.3389/fchem.2022.862483)

Supplementary Material

Supplementary Figure 1. The incorporation of acetyllysine (AcK) at individual lysine sites of AcnA and AcnB. SDS-PAGE (upper panels) and western blots (lower panels) of purified aconitases and their variants from BL21(DE3) cells. Lane 1, wild-type AcnA; lane 2, AcnA-164AcK; lane 3, AcnA-342AcK; lane 4, AcnA-482AcK; lane 5; AcnA-684AcK; lane 6, wild-type AcnB; lane 7, AcnB-77AcK; lane 8, AcnB-373AcK; lane 9, AcnB-396AcK; lane 10, AcnB-407AcK; lane 11, AcnB-539AcK; lane 12, AcnB-559AcK; lane 13, AcnB-567AcK; lane 14, AcnB-728AcK; lane 15, AcnB-758AcK; lane 16, AcnB-835AcK. For each lane, 2 mg of purified proteins were loaded. Anti-AcK: acetyllysine antibody.

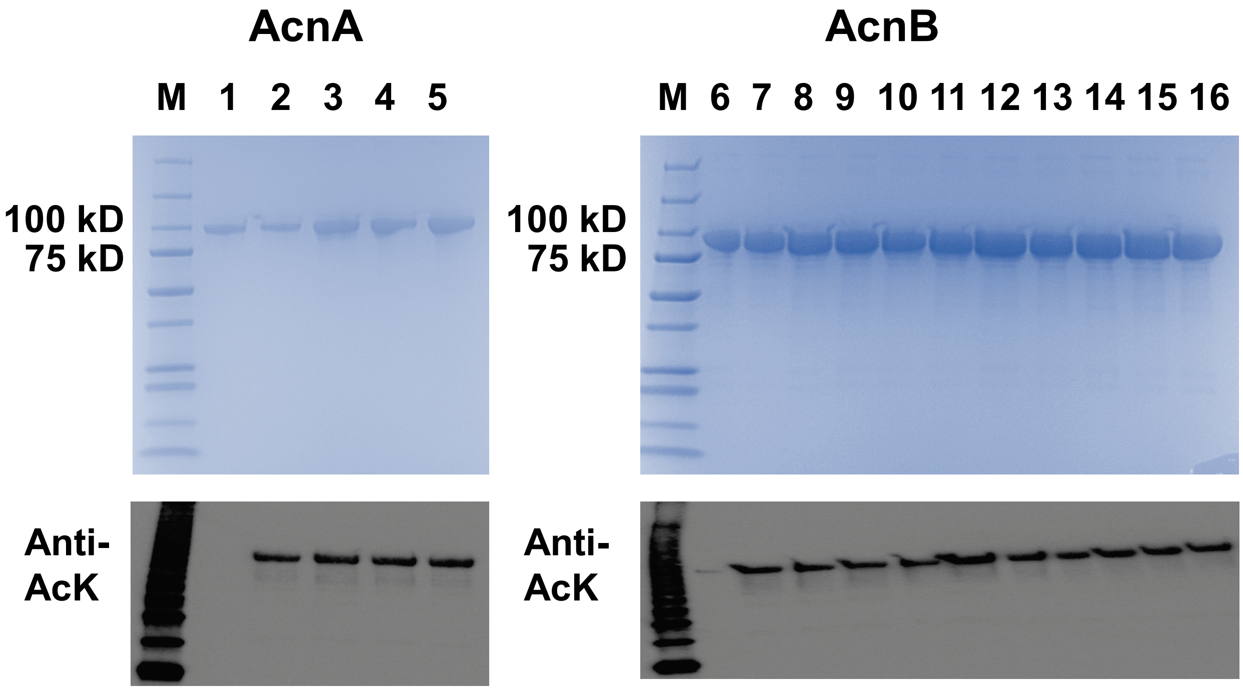

Supplementary Figure 2. LC-MS/MS analysis of AcnA 164-AcK. The tandem mass spectrum of the peptide (residues 162-169) WGKQAFSR from purified AcnA 164-AcK. K^{AC} denotes AcK incorporation. The partial sequence of the peptide containing the AcK can be read from the annotated a/b or y ion series. Matched peaks are in red.

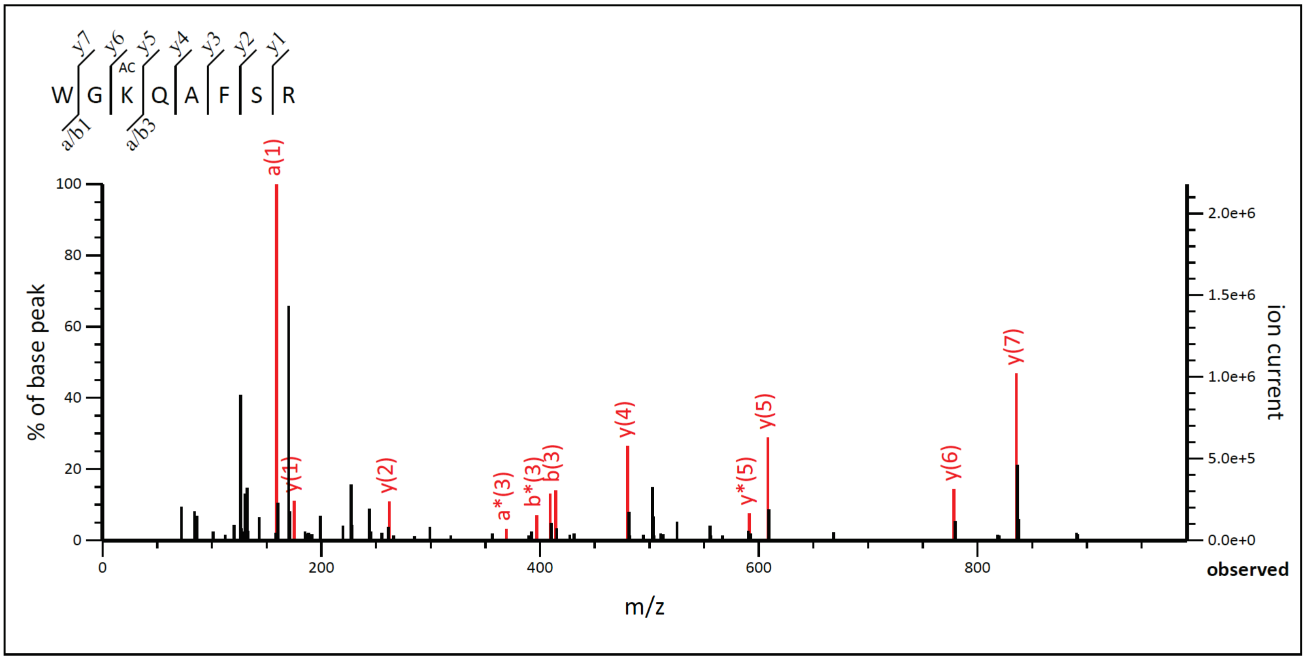

#	a	a++	a*	a*++	b	b++	b*	b*++	Seq.	y	y++	y*	y*++	#
1	**159.0917**	80.0495			187.0866	94.0469			W					8
2	216.1131	108.5602			244.1081	122.5577			G	***835.4421***	418.2247	818.4155	409.7114	7
3	386.2187	193.6130	369.1921	185.0997	**414.2136**	207.6104	397.1870	199.0972	K	***778.4206***	389.7139	761.3941	381.2007	6
4	514.2772	257.6423	497.2507	249.1290	542.2722	271.6397	525.2456	263.1264	Q	***608.3151***	304.6612	591.2885	296.1479	5
5	585.3144	293.1608	568.2878	284.6475	613.3093	307.1583	596.2827	298.6450	A	***480.2565***	240.6319	463.2300	232.1186	4
6	732.3828	366.6950	715.3562	358.1817	760.3777	380.6925	743.3511	372.1792	F	***409.2194***	205.1133	392.1928	196.6001	3
7	819.4148	410.2110	802.3883	401.6978	847.4097	424.2085	830.3832	415.6952	S	***262.1510***	131.5791	245.1244	123.0659	2
8									R	***175.1190***	88.0631	158.0924	79.5498	1

Supplementary Figure 3. LC-MS/MS analysis of AcnA 342-AcK. The tandem mass spectrum of the peptide (residues 333-345) SEDQVELVEKYAK from purified AcnA 342-AcK. K^{AC} denotes AcK incorporation. The partial sequence of the peptide containing the AcK can be read from the annotated a/b or y ion series. Matched peaks are in red.

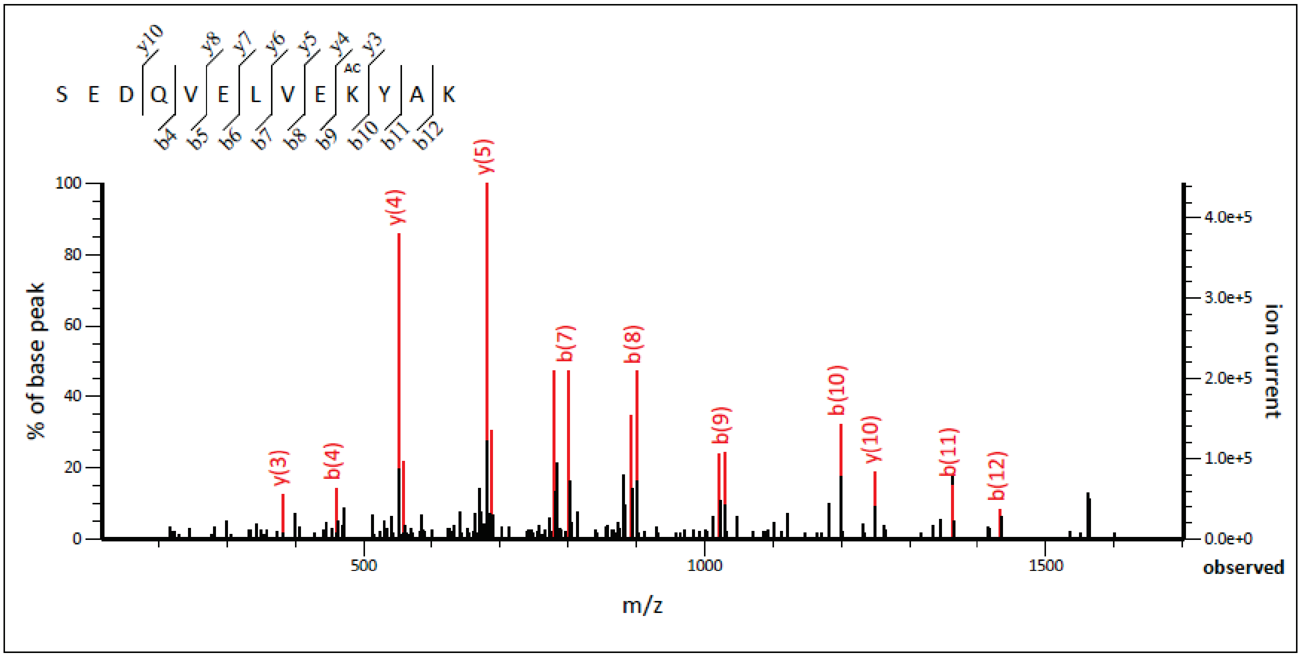

#	a	a*	b	b*	Seq.	y	y*	#
1	60.0444		88.0393		S			13
2	189.0870		217.0819		E	1492.7530	1475.7264	12
3	304.1139		332.1088		D	1363.7104	1346.6838	11
4	432.1725	415.1460	***460.1674***	443.1409	Q	**1248.6834**	1231.6569	10
5	531.2409	514.2144	***559.2358***	542.2093	V	1120.6249	1103.5983	9
6	660.2835	643.2570	***688.2784***	671.2519	E	**1021.5564**	1004.5299	8
7	773.3676	756.3410	***801.3625***	784.3359	L	**892.5138**	875.4873	7
8	872.4360	855.4094	***900.4309***	883.4044	V	**779.4298**	762.4032	6
9	1001.4786	984.4520	***1029.4735***	1012.4469	E	**680.3614**	663.3348	5
10	1171.5841	1154.5576	***1199.5790***	1182.5525	K	**551.3188**	534.2922	4
11	1334.6474	1317.6209	***1362.6424***	1345.6158	Y	**381.2132**	364.1867	3
12	1405.6846	1388.6580	***1433.6795***	1416.6529	A	218.1499	201.1234	2
13					K	147.1128	130.0863	1

Supplementary Figure 4. LC-MS/MS analysis of AcnA 482-AcK. The tandem mass spectrum of the peptide (residues 475-484) VVSDYLAKAK from purified AcnA 482-AcK. K^{AC} denotes AcK incorporation. The partial sequence of the peptide containing the AcK can be read from the annotated a/b or y ion series. Matched peaks are in red.

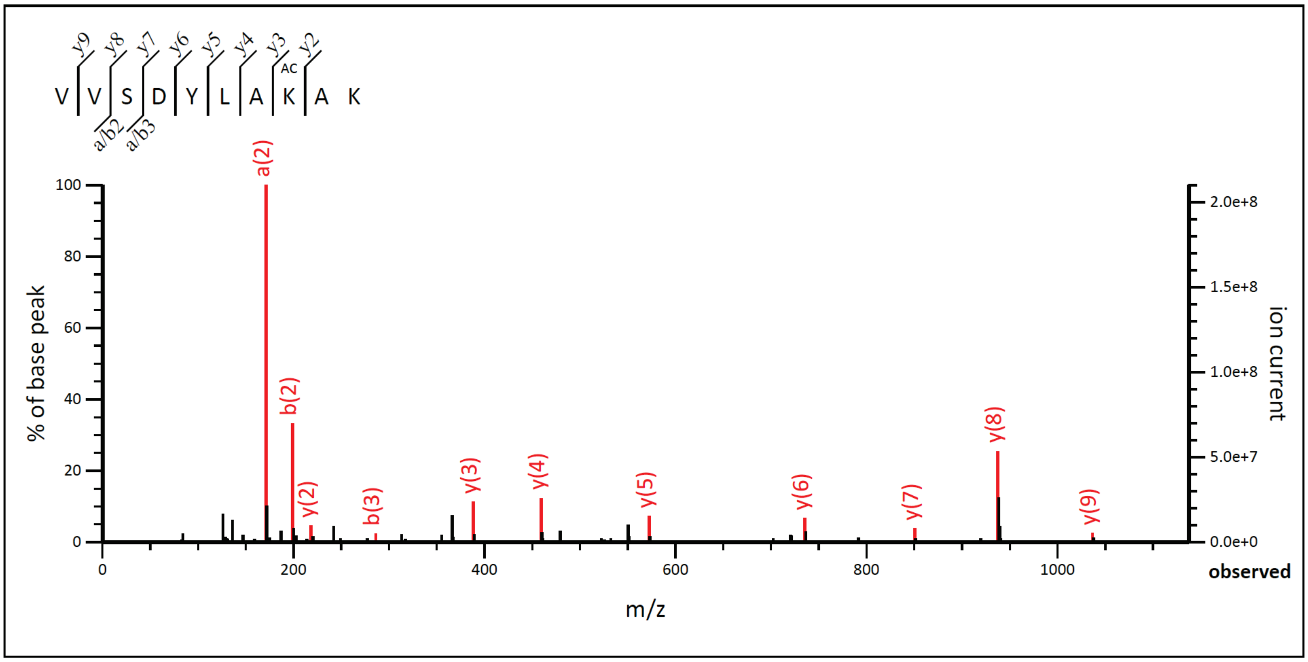

#	a	a^{++}	a*	a^{*++}	b	b^{++}	b*	b^{*++}	Seq.	y	y^{++}	y*	y^{*++}	#
1	72.0808	36.5440			100.0757	50.5415			V					10
2	**171.1492**	86.0782			**199.1441**	100.0757			V	***1036.5673***	518.7873	1019.5408	510.2740	9
3	258.1812	129.5942			**286.1761**	143.5917			S	***937.4989***	469.2531	920.4724	460.7398	8
4	373.2082	187.1077			401.2031	201.1052			D	***850.4669***	425.7371	833.4403	417.2238	7
5	536.2715	268.6394			564.2664	282.6368			Y	***735.4400***	368.2236	718.4134	359.7103	6
6	649.3556	325.1814			677.3505	339.1789			L	***572.3766***	286.6920	555.3501	278.1787	5
7	720.3927	360.7000			748.3876	374.6974			A	***459.2926***	230.1499	442.2660	221.6366	4
8	890.4982	445.7527	873.4716	437.2395	918.4931	459.7502	901.4666	451.2369	K	***388.2554***	194.6314	371.2289	186.1181	3
9	961.5353	481.2713	944.5088	472.7580	989.5302	495.2688	972.5037	486.7555	A	***218.1499***	109.5786	201.1234	101.0653	2
10									K	147.1128	74.0600	130.0863	65.5468	1

Supplementary Figure 5. LC-MS/MS analysis of AcnA 684-AcK. The tandem mass spectrum of the peptide (residues 664-691) ILAMLGDSVTTDHISPAGSIKPDSPAGR from purified AcnA 684-AcK. K^{AC} denotes AcK incorporation. The partial sequence of the peptide containing the AcK can be read from the annotated a/b or y ion series. Matched peaks are in red.

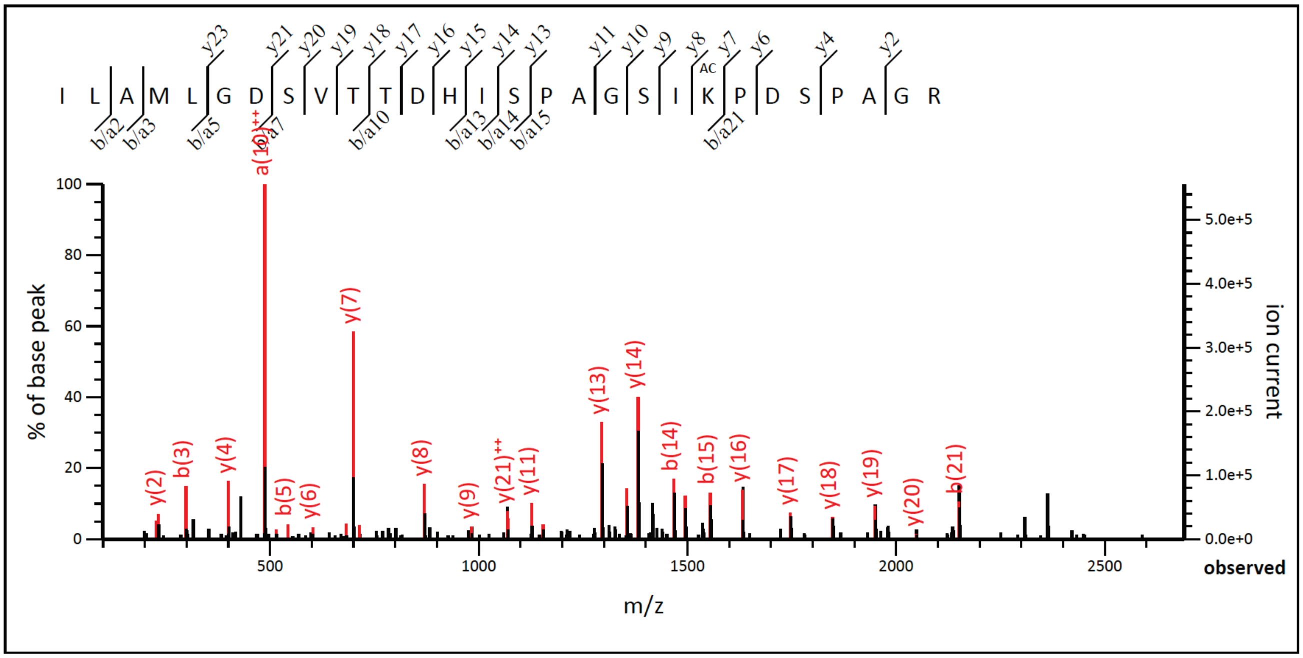

#	a	a^{++}	a*	a^{*++}	b	b^{++}	b*	b^{*++}	Seq.	y	y^{++}	y*	y^{*++}	#
1	86.0964	43.5519			114.0913	57.5493			I					28
2	199.1805	100.0939			227.1754	114.0913			L	2735.3669	1368.1871	2718.3403	1359.6738	27
3	270.2176	135.6124			298.2125	149.6099			A	2622.2828	1311.6450	2605.2563	1303.1318	26
4	401.2581	201.1327			429.2530	215.1301			M	2551.2457	1276.1265	2534.2191	1267.6132	25
5	514.3422	257.6747			542.3371	271.6722			L	2420.2052	1210.6062	2403.1787	1202.0930	24
6	571.3636	286.1854			599.3585	300.1829			G	2307.1211	1154.0642	2290.0946	1145.5509	23
7	686.3906	343.6989			714.3855	357.6964			D	2250.0997	1125.5535	2233.0731	1117.0402	22
8	773.4226	387.2149			801.4175	401.2124			S	2135.0727	1068.0400	2118.0462	1059.5267	21
9	872.4910	436.7491			900.4859	450.7466			V	2048.0407	1024.5240	2031.0142	1016.0107	20
10	973.5387	487.2730			1001.5336	501.2704			T	1948.9723	974.9898	1931.9457	966.4765	19
11	1074.5864	537.7968			1102.5813	551.7943			T	1847.9246	924.4659	1830.8981	915.9527	18
12	1189.6133	595.3103			1217.6082	609.3077			D	1746.8769	873.9421	1729.8504	865.4288	17
13	1326.6722	663.8397			1354.6671	677.8372			H	1631.8500	816.4286	1614.8234	807.9154	16
14	1439.7563	720.3818			1467.7512	734.3792			I	1494.7911	747.8992	1477.7645	739.3859	15
15	1526.7883	763.8978			1554.7832	777.8952			S	1381.7070	691.3571	1364.6805	682.8439	14
16	1623.8411	812.4242			1651.8360	826.4216			P	1294.6750	647.8411	1277.6484	639.3279	13
17	1694.8782	847.9427			1722.8731	861.9402			A	1197.6222	599.3148	1180.5957	590.8015	12
18	1751.8996	876.4535			1779.8946	890.4509			G	1126.5851	563.7962	1109.5586	555.2829	11
19	1838.9317	919.9695			1866.9266	933.9669			S	1069.5636	535.2855	1052.5371	526.7722	10
20	1952.0157	976.5115			1980.0107	990.5090			I	982.5316	491.7694	965.5051	483.2562	9
21	2122.1213	1061.5643	2105.0947	1053.0510	2150.1162	1075.5617	2133.0896	1067.0485	K	869.4476	435.2274	852.4210	426.7141	8
22	2219.1740	1110.0907	2202.1475	1101.5774	2247.1689	1124.0881	2230.1424	1115.5748	P	699.3420	350.1747	682.3155	341.6614	7
23	2334.2010	1167.6041	2317.1744	1159.0909	2362.1959	1181.6016	2345.1693	1173.0883	D	602.2893	301.6483	585.2627	293.1350	6
24	2421.2330	1211.1201	2404.2065	1202.6069	2449.2279	1225.1176	2432.2014	1216.6043	S	487.2623	244.1348	470.2358	235.6215	5
25	2518.2858	1259.6465	2501.2592	1251.1332	2546.2807	1273.6440	2529.2541	1265.1307	P	400.2303	200.6188	383.2037	192.1055	4
26	2589.3229	1295.1651	2572.2963	1286.6518	2617.3178	1309.1625	2600.2912	1300.6493	A	303.1775	152.0924	286.1510	143.5791	3
27	2646.3443	1323.6758	2629.3178	1315.1625	2674.3393	1337.6733	2657.3127	1329.1600	G	232.1404	116.5738	215.1139	108.0606	2
28									R	175.1190	88.0631	158.0924	79.5498	1

Supplementary Figure 6. LC-MS/MS analysis of AcnB 77-AcK. The tandem mass spectrum of the peptide (residues 74-85) GEAKSPLLTPEK from purified AcnB 77-AcK. K^{AC} denotes AcK incorporation. The partial sequence of the peptide containing the AcK can be read from the annotated a/b or y ion series. Matched peaks are in red.

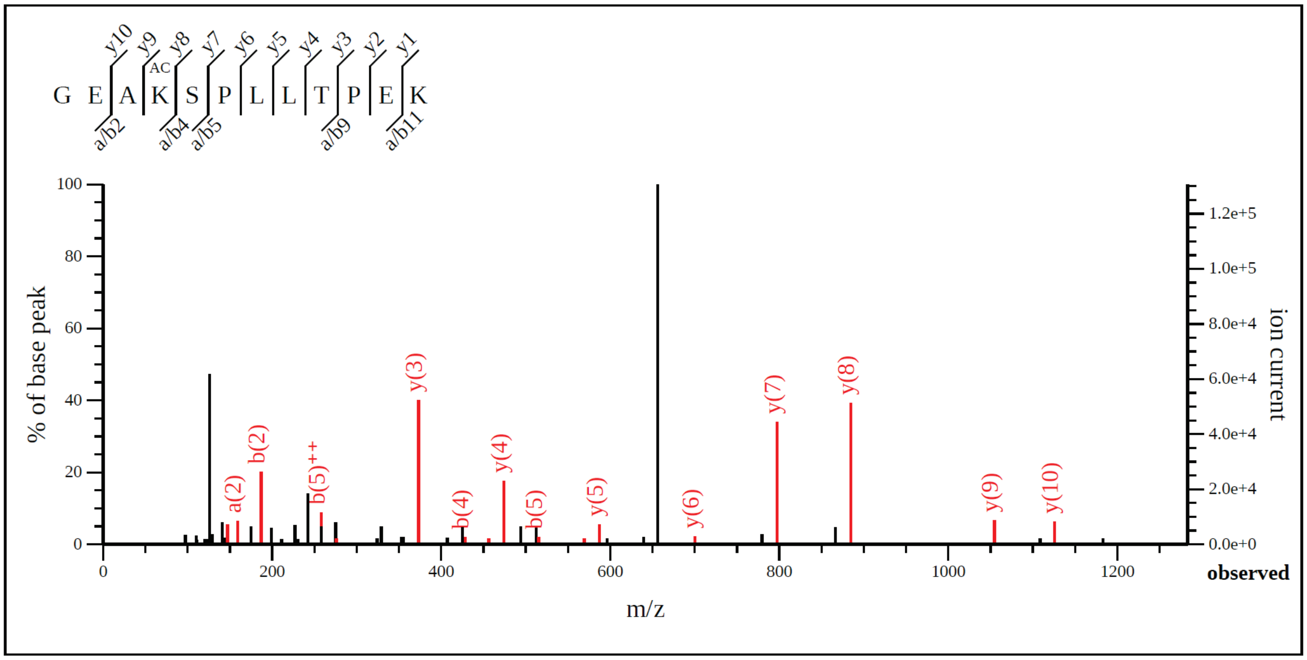

#	a	a++	a*	a*++	b	b++	b*	b*++	Seq.	y	y++	y*	y*++	#
1	30.0338	15.5206			58.0287	29.5180			G					12
2	159.0764	80.0418			187.0713	94.0393			E	1254.6940	627.8506	1237.6674	619.3374	11
3	230.1135	115.5604			258.1084	129.5579			A	1125.6514	563.3293	1108.6249	554.8161	10
4	400.2191	200.6132	383.1925	192.0999	428.2140	214.6106	411.1874	206.0974	K	1054.6143	527.8108	1037.5877	519.2975	9
5	487.2511	244.1292	470.2245	235.6159	515.2460	258.1266	498.2195	249.6134	S	884.5088	442.7580	867.4822	434.2447	8
6	584.3039	292.6556	567.2773	284.1423	612.2988	306.6530	595.2722	298.1397	P	797.4767	399.2420	780.4502	390.7287	7
7	697.3879	349.1976	680.3614	340.6843	725.3828	363.1951	708.3563	354.6818	L	700.4240	350.7156	683.3974	342.2023	6
8	810.4720	405.7396	793.4454	397.2264	838.4669	419.7371	821.4403	411.2238	L	587.3399	294.1736	570.3134	285.6603	5
9	911.5197	456.2635	894.4931	447.7502	939.5146	470.2609	922.4880	461.7477	T	474.2558	237.6316	457.2293	229.1183	4
10	1008.5724	504.7898	991.5459	496.2766	1036.5673	518.7873	1019.5408	510.2740	P	373.2082	187.1077	356.1816	178.5944	3
11	1137.6150	569.3111	1120.5885	560.7979	1165.6099	583.3086	1148.5834	574.7953	E	276.1554	138.5813	259.1288	130.0681	2
12									K	147.1128	74.0600	130.0863	65.5468	1

Supplementary Figure 7. LC-MS/MS analysis of AcnB 373-AcK. The tandem mass spectrum of the peptide (residues 371-380) QAKDVAESDR from purified AcnB 373-AcK. K^{AC} denotes AcK incorporation. The partial sequence of the peptide containing the AcK can be read from the annotated a/b or y ion series. Matched peaks are in red.

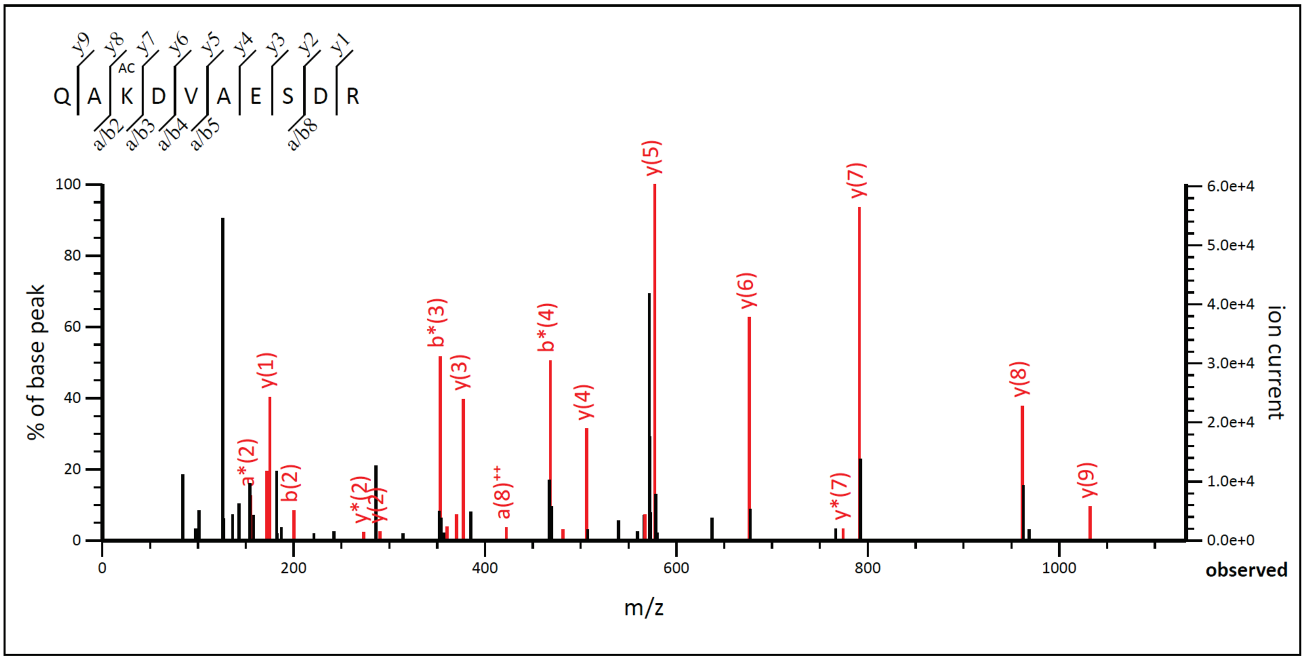

#	a	a++	a*	a*++	b	b++	b*	b*++	Seq.	y	y++	y*	y*++	#
1	101.0709	51.0391	84.0444	42.5258	129.0659	65.0366	112.0393	56.5233	Q					10
2	**172.1081**	86.5577	155.0815	78.0444	**200.1030**	100.5551	183.0764	92.0418	A	***1032.4956***	516.7515	1015.4691	508.2382	9
3	342.2136	171.6104	325.1870	163.0972	**370.2085**	185.6079	353.1819	177.0946	K	***961.4585***	**481.2329**	944.4320	472.7196	8
4	457.2405	229.1239	440.2140	220.6106	485.2354	243.1214	468.2089	234.6081	D	***791.3530***	396.1801	774.3264	387.6669	7
5	556.3089	278.6581	539.2824	270.1448	584.3039	292.6556	567.2773	284.1423	V	***676.3260***	338.6667	659.2995	330.1534	6
6	627.3461	314.1767	610.3195	305.6634	655.3410	328.1741	638.3144	319.6608	A	***577.2576***	289.1325	560.2311	280.6192	5
7	756.3886	378.6980	739.3621	370.1847	784.3836	392.6954	767.3570	384.1821	E	***506.2205***	253.6139	489.1940	245.1006	4
8	843.4207	**422.2140**	826.3941	413.7007	871.4156	436.2114	854.3890	427.6982	S	***377.1779***	189.0926	360.1514	180.5793	3
9	958.4476	479.7274	941.4211	471.2142	986.4425	493.7249	969.4160	485.2116	D	***290.1459***	145.5766	273.1193	137.0633	2
10									R	***175.1190***	88.0631	158.0924	79.5498	1

Supplementary Figure 8. LC-MS/MS analysis of AcnB 396-AcK. The tandem mass spectrum of the peptide (residues 392-399) ACGVKGIR from purified AcnB 396-AcK. K^{AC} denotes AcK incorporation. The partial sequence of the peptide containing the AcK can be read from the annotated a/b or y ion series. Matched peaks are in red.

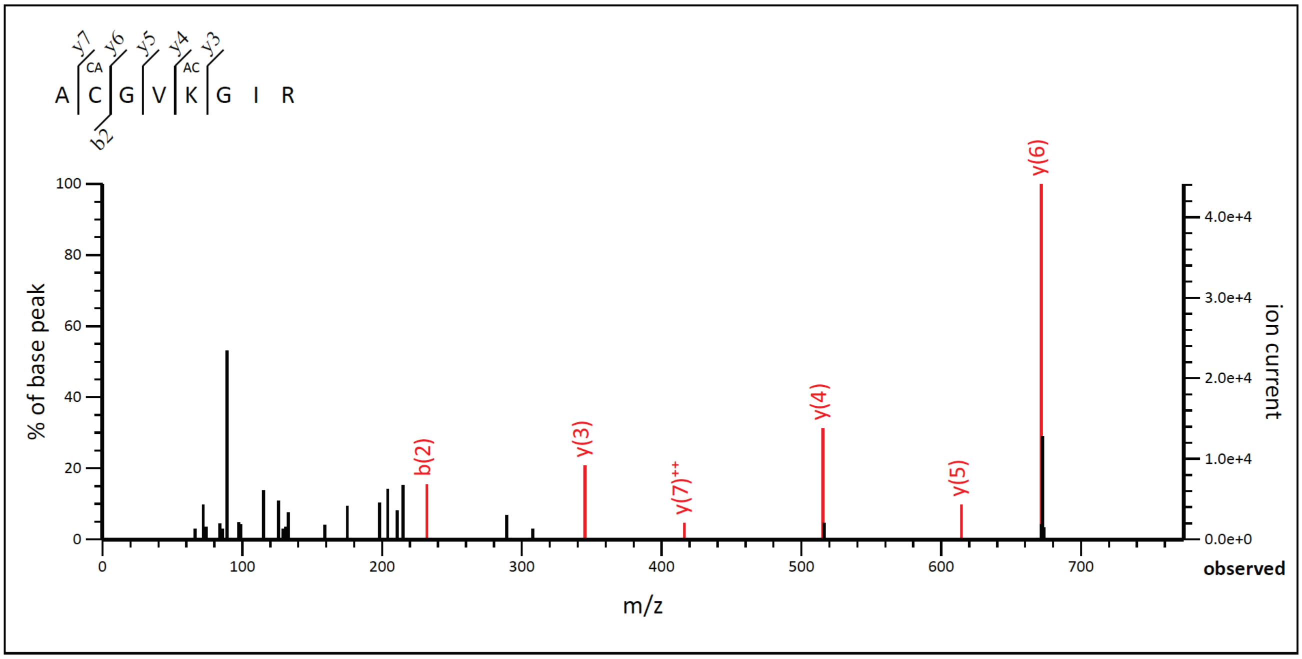

#	a	a^{++}	a*	a^{*++}	b	b^{++}	b*	b^{*++}	Seq.	y	y^{++}	y*	y^{*++}	#
1	44.0495	22.5284			72.0444	36.5258			A					8
2	204.0801	102.5437			**232.0750**	116.5412			C	831.4505	**416.2289**	814.4240	407.7156	7
3	261.1016	131.0544			289.0965	145.0519			G	***671.4199***	336.2136	654.3933	327.7003	6
4	360.1700	180.5886			388.1649	194.5861			V	***614.3984***	307.7028	597.3719	299.1896	5
5	530.2755	265.6414	513.2490	257.1281	558.2704	279.6389	541.2439	271.1256	K	***515.3300***	258.1686	498.3035	249.6554	4
6	587.2970	294.1521	570.2704	285.6389	615.2919	308.1496	598.2654	299.6363	G	***345.2245***	173.1159	328.1979	164.6026	3
7	700.3811	350.6942	683.3545	342.1809	728.3760	364.6916	711.3494	356.1784	I	288.2030	144.6051	271.1765	136.0919	2
8									R	175.1190	88.0631	158.0924	79.5498	1

Supplementary Figure 9. LC-MS/MS analysis of AcnB 407-AcK. The tandem mass spectrum of the peptide (residues 400-422) PGAYCEPKMTSVGSQDTTGPMTR from purified AcnB 407-AcK. K^{AC} denotes AcK incorporation. The partial sequence of the peptide containing the AcK can be read from the annotated a/b or y ion series. Matched peaks are in red.

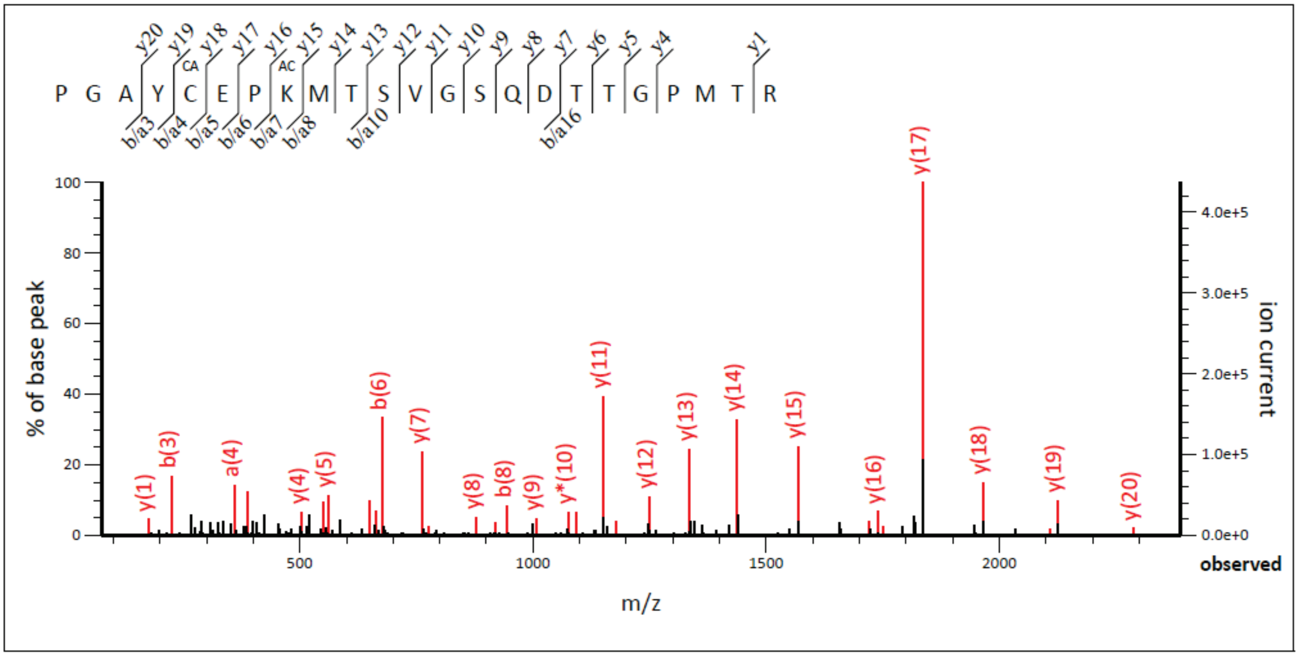

#	a	a^{++}	a*	a^{*++}	b	b^{++}	b*	b^{*++}	Seq.	y	y^{++}	y*	y^{*++}	#
1	70.0651	35.5362			98.0600	49.5337			P					23
2	127.0866	64.0469			155.0815	78.0444			G	2416.0578	1208.5325	2399.0312	1200.0192	22
3	198.1237	99.5655			**226.1186**	113.5629			A	2359.0363	1180.0218	2342.0097	1171.5085	21
4	**361.1870**	181.0972			**389.1819**	195.0946			Y	***2287.9992***	1144.5032	2270.9726	1135.9900	20
5	521.2177	261.1125			**549.2126**	275.1099			C	***2124.9359***	1062.9716	2107.9093	1054.4583	19
6	**650.2603**	325.6338			**678.2552**	339.6312			E	***1964.9052***	982.9562	1947.8787	974.4430	18
7	747.3130	374.1602			**775.3080**	388.1576			P	***1835.8626***	**918.4349**	1818.8361	909.9217	17
8	917.4186	459.2129	900.3920	450.6996	**945.4135**	473.2104	928.3869	464.6971	K	***1738.8098***	869.9086	1721.7833	861.3953	16
9	1048.4591	524.7332	1031.4325	516.2199	**1076.4540**	538.7306	1059.4274	530.2173	M	***1568.7043***	784.8558	1551.6778	776.3425	15
10	1149.5067	575.2570	1132.4802	566.7437	**1177.5016**	589.2545	1160.4751	580.7412	T	***1437.6638***	719.3356	1420.6373	710.8223	14
11	1236.5388	618.7730	1219.5122	610.2597	1264.5337	632.7705	1247.5071	624.2572	S	***1336.6162***	668.8117	1319.5896	660.2984	13
12	1335.6072	668.3072	1318.5806	659.7939	1363.6021	682.3047	1346.5755	673.7914	V	***1249.5841***	625.2957	1232.5576	616.7824	12
13	1392.6286	696.8180	1375.6021	688.3047	1420.6236	710.8154	1403.5970	702.3021	G	***1150.5157***	575.7615	1133.4892	567.2482	11
14	1479.6607	740.3340	1462.6341	731.8207	1507.6556	754.3314	1490.6290	745.8182	S	***1093.4943***	547.2508	1076.4677	538.7375	10
15	1607.7192	804.3633	1590.6927	795.8500	1635.7142	818.3607	1618.6876	809.8474	Q	***1006.4622***	503.7347	989.4357	495.2215	9
16	1722.7462	861.8767	1705.7196	853.3635	**1750.7411**	875.8742	1733.7146	867.3609	D	***878.4036***	439.7055	861.3771	431.1922	8
17	1823.7939	912.4006	1806.7673	903.8873	1851.7888	926.3980	1834.7622	917.8848	T	***763.3767***	382.1920	746.3502	373.6787	7
18	1924.8415	962.9244	1907.8150	954.4111	1952.8365	976.9219	1935.8099	968.4086	T	***662.3290***	331.6681	645.3025	323.1549	6
19	1981.8630	991.4351	1964.8365	982.9219	2009.8579	1005.4326	1992.8314	996.9193	G	***561.2813***	281.1443	544.2548	272.6310	5
20	2078.9158	1039.9615	2061.8892	1031.4482	2106.9107	1053.9590	2089.8841	1045.4457	P	***504.2599***	252.6336	487.2333	244.1203	4
21	2209.9563	1105.4818	2192.9297	1096.9685	2237.9512	1119.4792	2220.9246	1110.9659	M	407.2071	204.1072	390.1806	195.5939	3
22	2311.0039	1156.0056	2293.9774	1147.4923	2338.9988	1170.0031	2321.9723	1161.4898	T	276.1666	138.5870	259.1401	130.0737	2
23									R	***175.1190***	88.0631	158.0924	79.5498	1

Supplementary Figure 10. LC-MS/MS analysis of AcnB 539-AcK. The tandem mass spectrum of the peptide (residues 536-547) FEGKMQPGITLR from purified AcnB 539-AcK. K^{AC} denotes AcK incorporation. The partial sequence of the peptide containing the AcK can be read from the annotated a/b or y ion series. Matched peaks are in red.

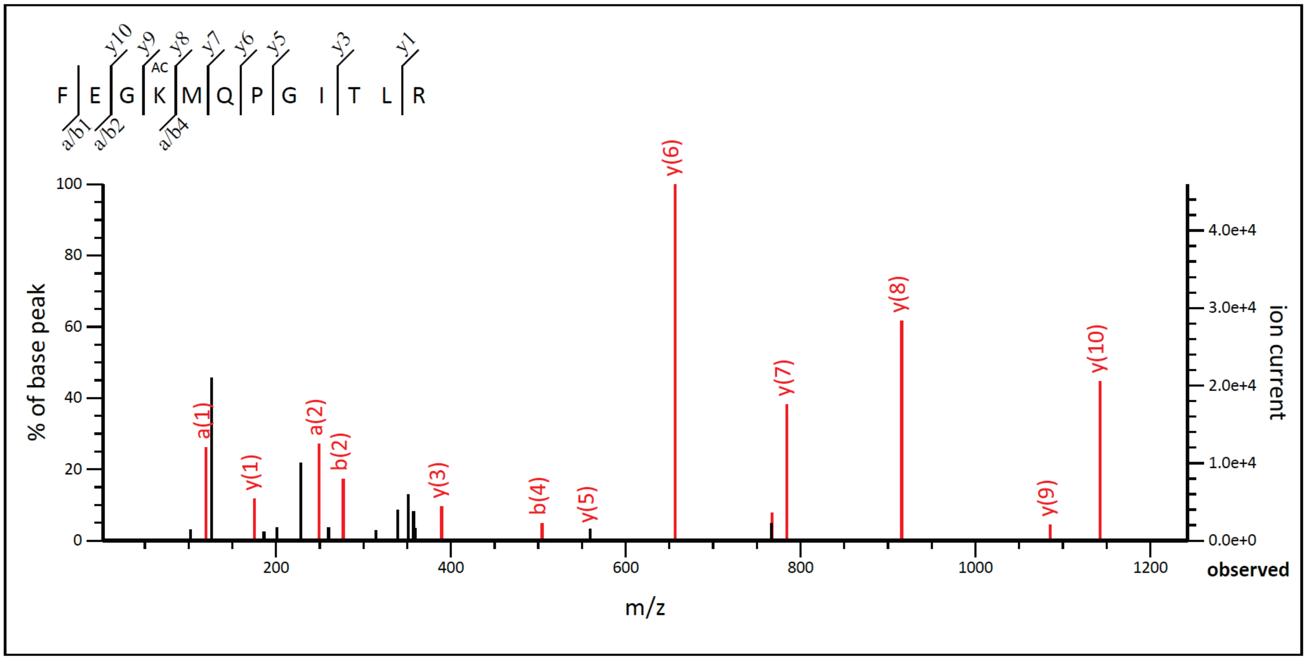

#	a	a++	a*	a*++	b	b++	b*	b*++	Seq.	y	y++	y*	y*++	#
1	**120.0808**	60.5440			148.0757	74.5415			F					12
2	**249.1234**	125.0653			**277.1183**	139.0628			E	1271.6776	636.3425	1254.6511	627.8292	11
3	306.1448	153.5761			334.1397	167.5735			G	***1142.6350***	571.8212	1125.6085	563.3079	10
4	476.2504	238.6288	459.2238	230.1155	**504.2453**	252.6263	487.2187	244.1130	K	***1085.6136***	543.3104	1068.5870	534.7972	9
5	607.2908	304.1491	590.2643	295.6358	635.2858	318.1465	618.2592	309.6332	M	***915.5080***	458.2577	898.4815	449.7444	8
6	735.3494	368.1783	718.3229	359.6651	763.3443	382.1758	746.3178	373.6625	Q	***784.4676***	392.7374	767.4410	384.2241	7
7	832.4022	416.7047	815.3756	408.1915	860.3971	430.7022	843.3706	422.1889	P	***656.4090***	328.7081	639.3824	320.1949	6
8	889.4237	445.2155	872.3971	436.7022	917.4186	459.2129	900.3920	450.6996	G	***559.3562***	280.1817	542.3297	271.6685	5
9	1002.5077	501.7575	985.4812	493.2442	1030.5026	515.7550	1013.4761	507.2417	I	502.3348	251.6710	485.3082	243.1577	4
10	1103.5554	552.2813	1086.5288	543.7681	1131.5503	566.2788	1114.5238	557.7655	T	***389.2507***	195.1290	372.2241	186.6157	3
11	1216.6395	608.8234	1199.6129	600.3101	1244.6344	622.8208	1227.6078	614.3075	L	288.2030	144.6051	271.1765	136.0919	2
12									R	***175.1190***	88.0631	158.0924	79.5498	1

Supplementary Figure 11. LC-MS/MS analysis of AcnB 559-AcK. The tandem mass spectrum of the peptide (residues 548-567) DLVHAIPLYAIKQGLLTVEK from purified AcnB 559-AcK. K^{AC} denotes AcK incorporation. The partial sequence of the peptide containing the AcK can be read from the annotated a/b or y ion series. Matched peaks are in red.

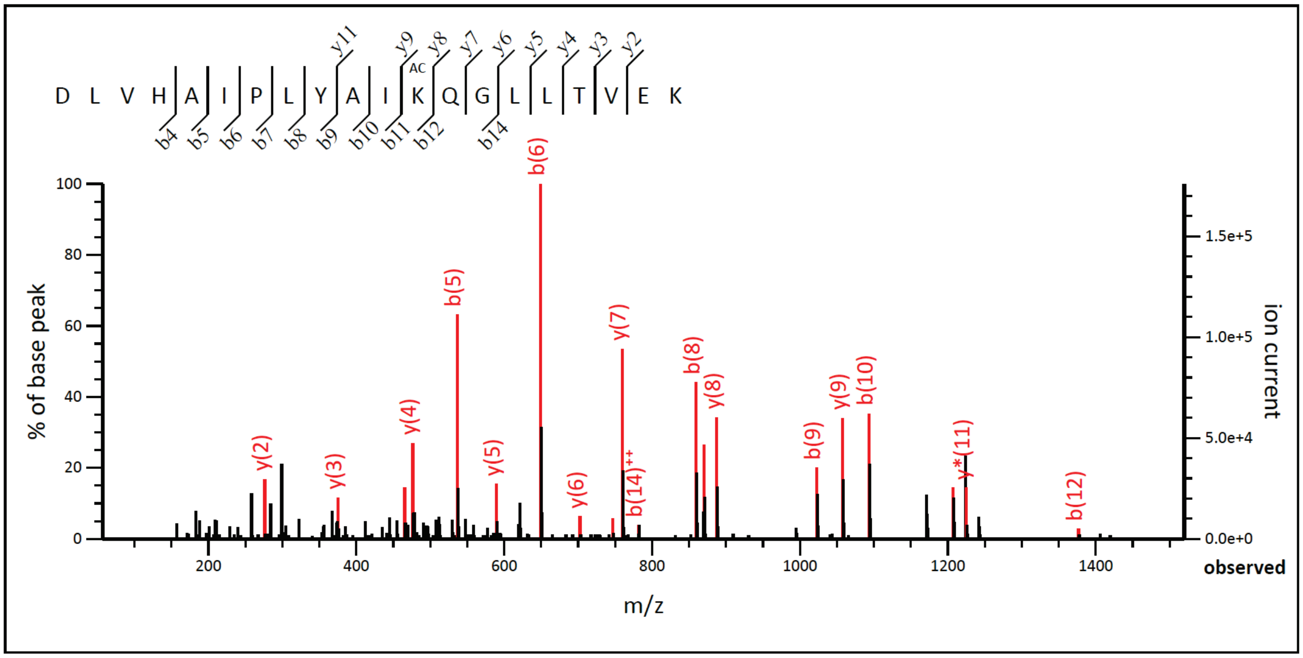

#	a	a^{++}	a*	a^{*++}	b	b^{++}	b*	b^{*++}	Seq.	y	y^{++}	y*	y^{*++}	#
1	88.0393	44.5233			116.0342	58.5207			D					20
2	201.1234	101.0653			229.1183	115.0628			L	2148.2791	1074.6432	2131.2525	1066.1299	19
3	300.1918	150.5995			328.1867	164.5970			V	2035.1950	1018.1012	2018.1685	1009.5879	18
4	437.2507	219.1290			***465.2456***	233.1264			H	1936.1266	968.5669	1919.1001	960.0537	17
5	508.2878	254.6475			***536.2827***	268.6450			A	1799.0677	900.0375	1782.0412	891.5242	16
6	621.3719	311.1896			***649.3668***	325.1870			I	1728.0306	864.5189	1711.0040	856.0057	15
7	718.4246	359.7160			***746.4196***	373.7134			P	1614.9465	807.9769	1597.9200	799.4636	14
8	831.5087	416.2580			***859.5036***	430.2554			L	1517.8938	**759.4505**	1500.8672	750.9372	13
9	994.5720	497.7897			***1022.5669***	511.7871			Y	1404.8097	702.9085	1387.7831	694.3952	12
10	1065.6091	533.3082			***1093.6041***	547.3057			A	1241.7464	621.3768	1224.7198	612.8635	11
11	1178.6932	589.8502			***1206.6881***	603.8477			I	1170.7093	585.8583	1153.6827	577.3450	10
12	1348.7987	674.9030	1331.7722	666.3897	***1376.7937***	688.9005	1359.7671	680.3872	K	***1057.6252***	529.3162	1040.5986	520.8030	9
13	1476.8573	738.9323	1459.8308	730.4190	1504.8522	752.9298	1487.8257	744.4165	Q	***887.5197***	444.2635	870.4931	435.7502	8
14	1533.8788	767.4430	1516.8522	758.9298	1561.8737	**781.4405**	1544.8471	772.9272	G	***759.4611***	380.2342	742.4345	371.7209	7
15	1646.9628	823.9851	1629.9363	815.4718	1674.9578	837.9825	1657.9312	829.4692	L	***702.4396***	351.7234	685.4131	343.2102	6
16	1760.0469	880.5271	1743.0204	872.0138	1788.0418	894.5245	1771.0153	886.0113	L	***589.3556***	295.1814	572.3290	286.6681	5
17	1861.0946	931.0509	1844.0680	922.5377	1889.0895	945.0484	1872.0629	936.5351	T	***476.2715***	238.6394	459.2449	230.1261	4
18	1960.1630	980.5851	1943.1364	972.0719	1988.1579	994.5826	1971.1314	986.0693	V	***375.2238***	188.1155	358.1973	179.6023	3
19	2089.2056	1045.1064	2072.1790	1036.5932	2117.2005	1059.1039	2100.1740	1050.5906	E	***276.1554***	138.5813	259.1288	130.0681	2
20									K	147.1128	74.0600	130.0863	65.5468	1

Supplementary Figure 12. LC-MS/MS analysis of AcnB 567-AcK. The tandem mass spectrum of the peptide (residues 560-568) QGLLTVEKK from purified AcnB 567-AcK. K^{AC} denotes AcK incorporation. The partial sequence of the peptide containing the AcK can be read from the annotated a/b or y ion series. Matched peaks are in red.

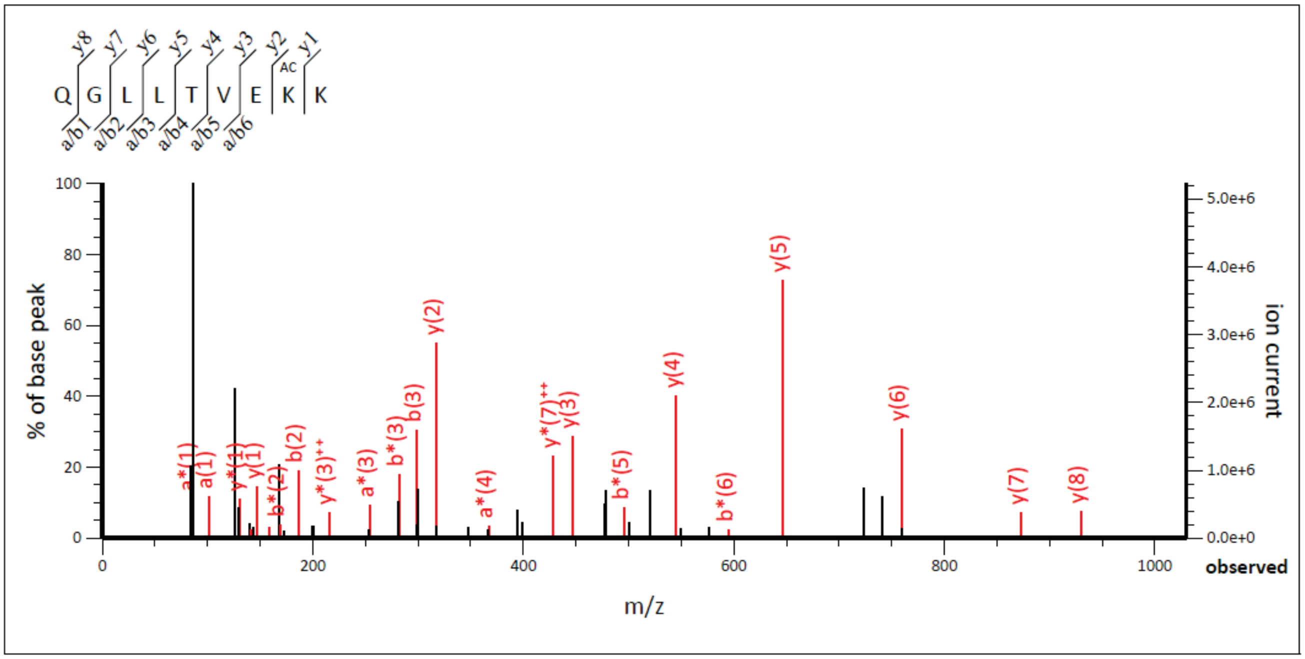

#	a	a^{++}	a*	a^{*++}	b	b^{++}	b*	b^{*++}	Seq.	y	y^{++}	y*	y^{*++}
1	**101.0709**	51.0391	**84.0444**	42.5258	**129.0659**	65.0366	112.0393	56.5233	Q				
2	**158.0924**	79.5498	**141.0659**	71.0366	**186.0873**	93.5473	**169.0608**	85.0340	G	**929.5666**	465.2869	912.5401	456.7737
3	271.1765	136.0919	**254.1499**	127.5786	**299.1714**	150.0893	**282.1448**	141.5761	L	**872.5451**	436.7762	855.5186	**428.2629**
4	384.2605	192.6339	**367.2340**	184.1206	412.2554	206.6314	395.2289	198.1181	L	**759.4611**	380.2342	742.4345	371.7209
5	485.3082	243.1577	468.2817	234.6445	513.3031	257.1552	**496.2766**	248.6419	T	**646.3770**	323.6921	629.3505	315.1789
6	584.3766	292.6920	567.3501	284.1787	612.3715	306.6894	**595.3450**	298.1761	V	**545.3293**	273.1683	528.3028	264.6550
7	713.4192	357.2132	696.3927	348.7000	741.4141	371.2107	724.3876	362.6974	E	**446.2609**	223.6341	429.2344	**215.1208**
8	883.5247	442.2660	866.4982	433.7527	911.5197	456.2635	894.4931	447.7502	K	**317.2183**	159.1128	300.1918	150.5995
9									K	**147.1128**	74.0600	**130.0863**	65.5468

Supplementary Figure 13. LC-MS/MS analysis of AcnB 728-AcK. The tandem mass spectrum of the peptide (residues 723-734) LLDAHKGQLPTR from purified AcnB 728-AcK. K^{AC} denotes AcK incorporation. The partial sequence of the peptide containing the AcK can be read from the annotated a/b or y ion series. Matched peaks are in red.

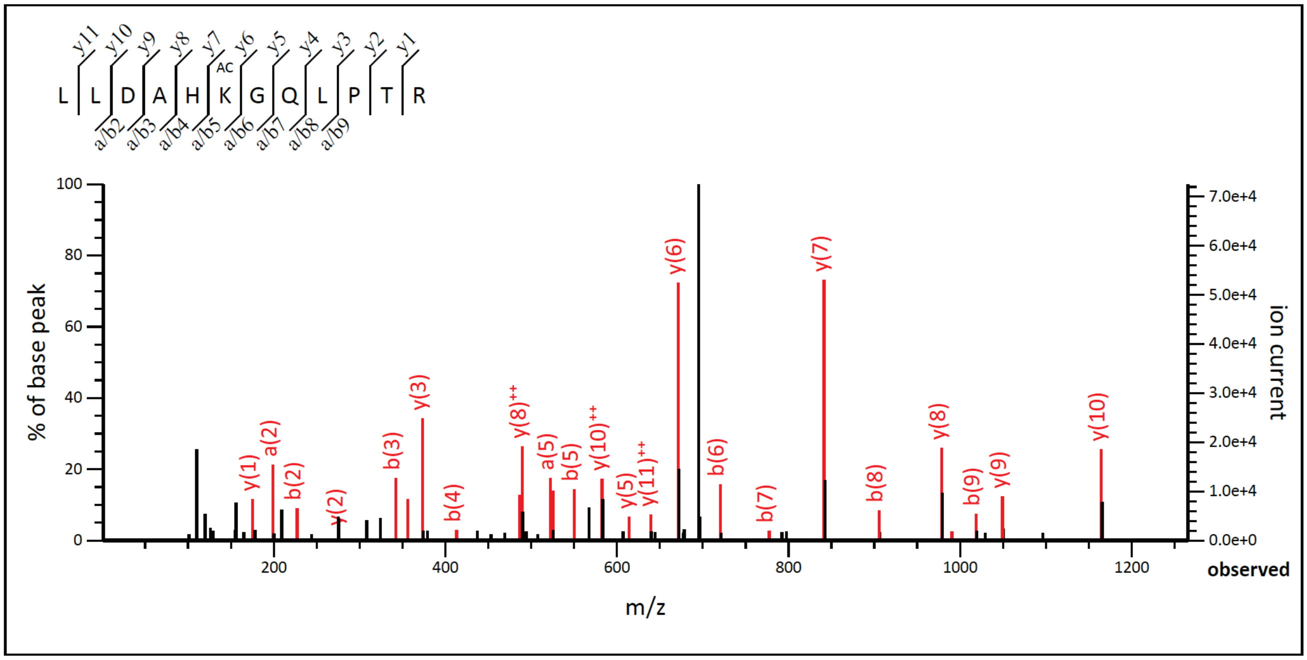

#	a	a^{++}	a*	a^{*++}	b	b^{++}	b*	b^{*++}	Seq.	y	y^{++}	y*	y^{*++}	#
1	86.0964	43.5519			114.0913	57.5493			L					12
2	**199.1805**	100.0939			**227.1754**	114.0913			L	1277.6961	**639.3517**	1260.6695	630.8384	11
3	314.2074	157.6074			**342.2023**	171.6048			D	***1164.6120***	**582.8096**	1147.5854	574.2964	10
4	385.2445	193.1259			**413.2395**	207.1234			A	***1049.5851***	**525.2962**	1032.5585	516.7829	9
5	**522.3035**	261.6554			**550.2984**	275.6528			H	***978.5479***	**489.7776**	961.5214	481.2643	8
6	692.4090	346.7081	675.3824	338.1949	**720.4039**	360.7056	703.3774	352.1923	K	***841.4890***	421.2482	824.4625	412.7349	7
7	749.4305	375.2189	732.4039	366.7056	**777.4254**	389.2163	760.3988	380.7030	G	***671.3835***	336.1954	654.3570	327.6821	6
8	877.4890	439.2482	860.4625	430.7349	**905.4839**	453.2456	888.4574	444.7323	Q	***614.3620***	307.6847	597.3355	299.1714	5
9	**990.5731**	495.7902	973.5465	487.2769	**1018.5680**	509.7876	1001.5415	501.2744	L	***486.3035***	243.6554	469.2769	235.1421	4
10	1087.6259	544.3166	1070.5993	535.8033	1115.6208	558.3140	1098.5942	549.8007	P	***373.2194***	187.1133	356.1928	178.6001	3
11	1188.6735	594.8404	1171.6470	586.3271	1216.6685	608.8379	1199.6419	600.3246	T	***276.1666***	138.5870	259.1401	130.0737	2
12									R	***175.1190***	88.0631	158.0924	79.5498	1

Supplementary Figure 14. LC-MS/MS analysis of AcnB 759-AcK. The tandem mass spectrum of the peptide (residues 743-763) MDAAQLTEEGYYSVFGKSGAR from purified AcnB 759-AcK. K^{AC} denotes AcK incorporation. The partial sequence of the peptide containing the AcK can be read from the annotated a/b or y ion series. Matched peaks are in red.

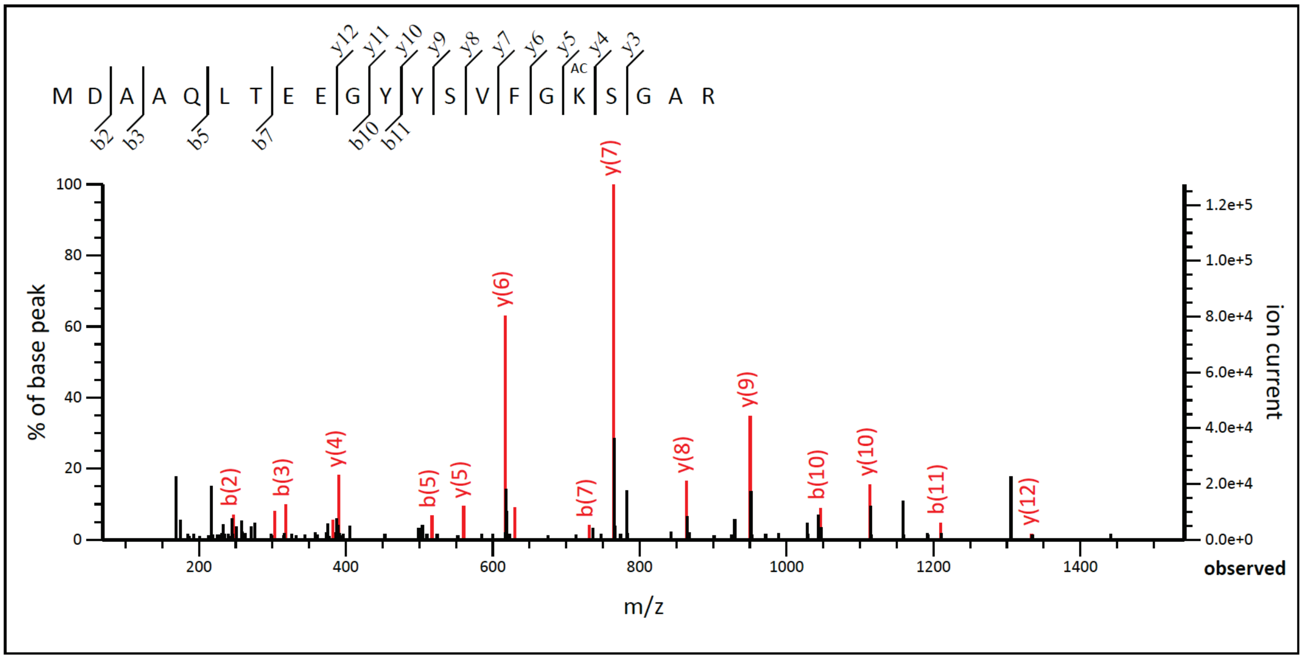

#	a	a^{++}	a*	a^{*++}	b	b^{++}	b*	b^{*++}	Seq.	y	y^{++}	y*	y^{*++}	#
1	104.0528	52.5301			132.0478	66.5275			M					21
2	219.0798	110.0435			247.0747	124.0410			D	2191.0302	1096.0187	2174.0037	1087.5055	20
3	290.1169	145.5621			318.1118	159.5595			A	2076.0033	1038.5053	2058.9767	1029.9920	19
4	361.1540	181.0806			389.1489	195.0781			A	2004.9661	1002.9867	1987.9396	994.4734	18
5	489.2126	245.1099	472.1860	236.5967	517.2075	259.1074	500.1810	250.5941	Q	1933.9290	967.4682	1916.9025	958.9549	17
6	602.2967	301.6520	585.2701	293.1387	630.2916	315.6494	613.2650	307.1362	L	1805.8705	903.4389	1788.8439	894.9256	16
7	703.3443	352.1758	686.3178	343.6625	731.3393	366.1733	714.3127	357.6600	T	1692.7864	846.8968	1675.7598	838.3836	15
8	832.3869	416.6971	815.3604	408.1838	860.3818	430.6946	843.3553	422.1813	E	1591.7387	796.3730	1574.7122	787.8597	14
9	961.4295	481.2184	944.4030	472.7051	989.4244	495.2159	972.3979	486.7026	E	1462.6961	731.8517	1445.6696	723.3384	13
10	1018.4510	509.7291	1001.4244	501.2159	1046.4459	523.7266	1029.4194	515.2133	G	1333.6535	667.3304	1316.6270	658.8171	12
11	1181.5143	591.2608	1164.4878	582.7475	1209.5092	605.2583	1192.4827	596.7450	Y	1276.6321	638.8197	1259.6055	630.3064	11
12	1344.5776	672.7925	1327.5511	664.2792	1372.5726	686.7899	1355.5460	678.2766	Y	1113.5687	557.2880	1096.5422	548.7747	10
13	1431.6097	716.3085	1414.5831	707.7952	1459.6046	730.3059	1442.5780	721.7927	S	950.5054	475.7563	933.4789	467.2431	9
14	1530.6781	765.8427	1513.6515	757.3294	1558.6730	779.8401	1541.6465	771.3269	V	863.4734	432.2403	846.4468	423.7271	8
15	1677.7465	839.3769	1660.7200	830.8636	1705.7414	853.3743	1688.7149	844.8611	F	764.4050	382.7061	747.3784	374.1928	7
16	1734.7680	867.8876	1717.7414	859.3743	1762.7629	881.8851	1745.7363	873.3718	G	617.3366	309.1719	600.3100	300.6586	6
17	1904.8735	952.9404	1887.8469	944.4271	1932.8684	966.9378	1915.8419	958.4246	K	560.3151	280.6612	543.2885	272.1479	5
18	1991.9055	996.4564	1974.8790	987.9431	2019.9004	1010.4539	2002.8739	1001.9406	S	390.2096	195.6084	373.1830	187.0951	4
19	2048.9270	1024.9671	2031.9004	1016.4539	2076.9219	1038.9646	2059.8954	1030.4513	G	303.1775	152.0924	286.1510	143.5791	3
20	2119.9641	1060.4857	2102.9376	1051.9724	2147.9590	1074.4831	2130.9325	1065.9699	A	246.1561	123.5817	229.1295	115.0684	2
21									R	175.1190	88.0631	158.0924	79.5498	1

Supplementary Figure 15. LC-MS/MS analysis of AcnB 835-AcK. The tandem mass spectrum of the peptide (residues 820-842) LPTPEEYQTYVAQVDKTAVDTYR from purified AcnB 835-AcK. K^{AC} denotes AcK incorporation. The partial sequence of the peptide containing the AcK can be read from the annotated a/b or y ion series. Matched peaks are in red.

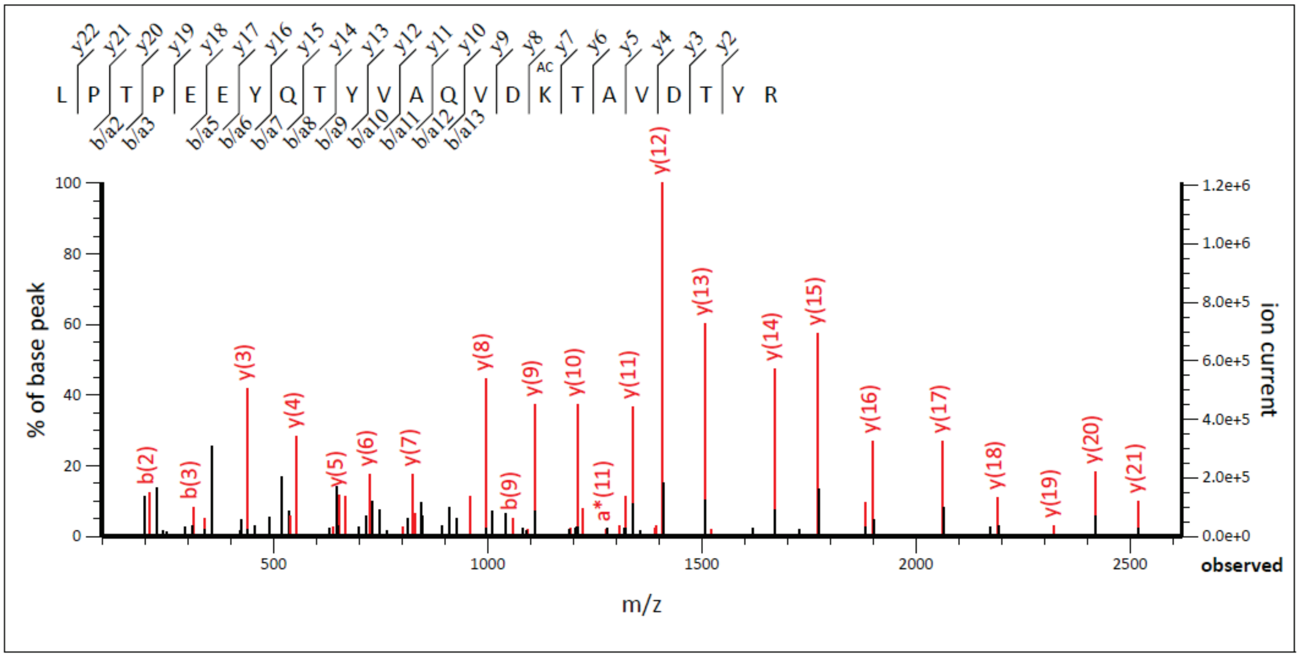

#	a	a++	a*	a*++	b	b++	b*	b*++	Seq.	y	y++	y*	y*++	#
1	86.0964	43.5519			114.0913	57.5493			L					23
2	183.1492	92.0782			**211.1441**	106.0757			P	2616.2464	**1308.6268**	2599.2199	1300.1136	22
3	284.1969	142.6021			**312.1918**	156.5995			T	**2519.1936**	1260.1005	2502.1671	1251.5872	21
4	381.2496	191.1285			409.2445	205.1259			P	**2418.1460**	1209.5766	2401.1194	1201.0633	20
5	510.2922	255.6498			**538.2871**	269.6472			E	**2321.0932**	1161.0502	2304.0667	1152.5370	19
6	**639.3348**	320.1710			**667.3297**	334.1685			E	**2192.0506**	1096.5289	2175.0241	1088.0157	18
7	**802.3981**	401.7027			**830.3931**	415.7002			Y	**2063.0080**	1032.0076	2045.9815	1023.4944	17
8	930.4567	465.7320	913.4302	457.2187	**958.4516**	479.7295	941.4251	471.2162	Q	**1899.9447**	950.4760	**1882.9181**	941.9627	16
9	1031.5044	516.2558	1014.4779	507.7426	**1059.4993**	530.2533	1042.4728	521.7400	T	**1771.8861**	886.4467	1754.8596	877.9334	15
10	**1194.5677**	597.7875	1177.5412	589.2742	**1222.5626**	611.7850	1205.5361	603.2717	Y	**1670.8384**	835.9229	1653.8119	827.4096	14
11	1293.6361	647.3217	**1276.6096**	638.8084	**1321.6311**	661.3192	1304.6045	652.8059	V	**1507.7751**	754.3912	1490.7486	745.8779	13
12	1364.6733	682.8403	1347.6467	674.3270	**1392.6682**	696.8377	1375.6416	688.3245	A	**1408.7067**	704.8570	**1391.6801**	696.3437	12
13	1492.7318	746.8696	1475.7053	738.3563	**1520.7268**	760.8670	1503.7002	752.3537	Q	**1337.6696**	669.3384	**1320.6430**	660.8251	11
14	1591.8003	796.4038	1574.7737	787.8905	1619.7952	810.4012	1602.7686	801.8879	V	**1209.6110**	605.3091	1192.5844	596.7959	10
15	1706.8272	853.9172	1689.8006	845.4040	1734.8221	867.9147	1717.7956	859.4014	D	**1110.5426**	555.7749	**1093.5160**	547.2617	9
16	1876.9327	938.9700	1859.9062	930.4567	1904.9276	952.9675	1887.9011	944.4542	K	**995.5156**	498.2615	978.4891	489.7482	8
17	1977.9804	989.4938	1960.9539	980.9806	2005.9753	1003.4913	1988.9488	994.9780	T	**825.4101**	413.2087	808.3836	404.6954	7
18	2049.0175	1025.0124	2031.9910	1016.4991	2077.0124	1039.0099	2059.9859	1030.4966	A	**724.3624**	362.6849	707.3359	354.1716	6
19	2148.0859	1074.5466	2131.0594	1066.0333	2176.0808	1088.5441	2159.0543	1080.0308	V	**653.3253**	327.1663	636.2988	318.6530	5
20	2263.1129	1132.0601	2246.0863	1123.5468	2291.1078	1146.0575	2274.0812	1137.5443	D	**554.2569**	277.6321	537.2304	269.1188	4
21	2364.1606	1182.5839	2347.1340	1174.0706	2392.1555	1196.5814	2375.1289	1188.0681	T	**439.2300**	220.1186	422.2034	211.6053	3
22	2527.2239	1264.1156	2510.1973	1255.6023	2555.2188	1278.1130	2538.1922	1269.5998	Y	**338.1823**	169.5948	321.1557	161.0815	2
23									R	175.1190	88.0631	158.0924	79.5498	1

Supplementary Figure 16. The full image of western blots for AcP-acetylation experiments in Figure 2A.

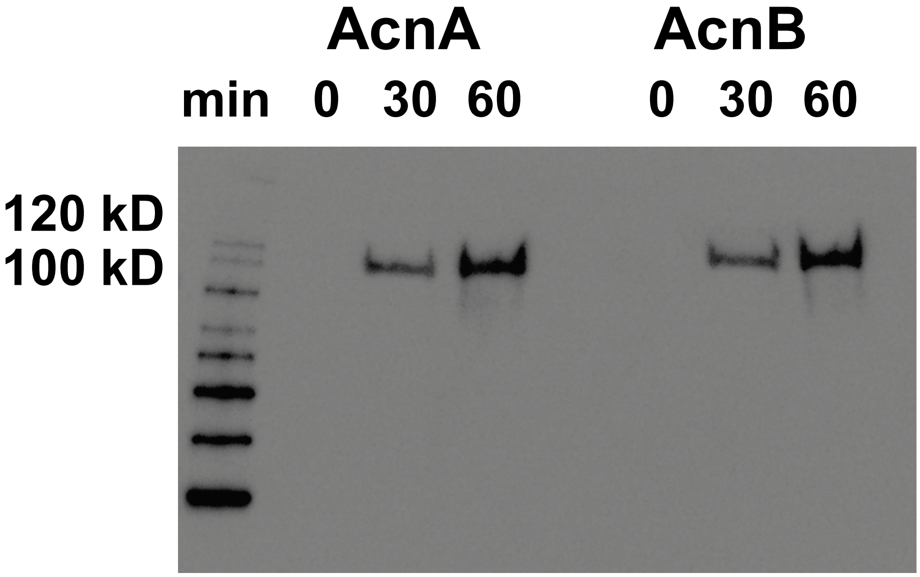

Supplementary Figure 17. The full image of western blots for CobB-deacetylation experiments in Figure 3A.

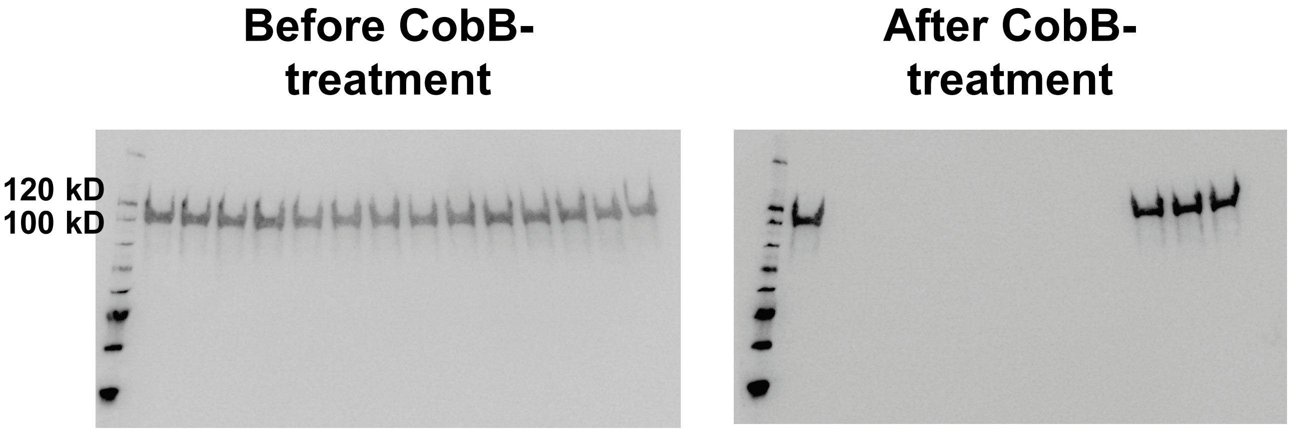

Supplementary Figure 18. Mapping of CobB-sensitive acetylated lysine sites on AcnA and AcnB structures. The structure of AcnA is generated by homology modeling with human cAcn as the template (PDB ID: 2B3Y). The structure of AcnB is from PDB ID: 1L5J.

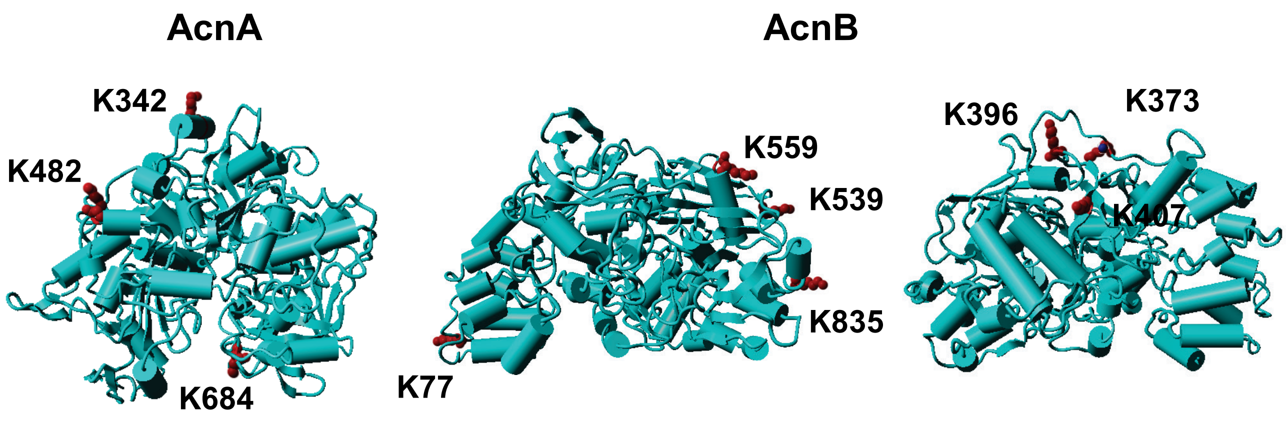

Supplement: Supplementary file 1 [file DataSheet1.PDF]
